# Unraveling the Synergistic Inhibition of Human Maltase–Glucoamylase by Baicalein and Acarbose: Integrated Pharmacodynamics and Computational Insights

**DOI:** 10.3390/ph19081215

**Published:** 2026-08-01

**Authors:** Xiaoshi He, Xia Li, Danyang Zhang, Hui Jiang, Yuesheng Dong

**Affiliations:** 1 School of Bioengineering, Dalian University of Technology, Dalian 116024, China; syingjjip@163.com (X.H.); zhangdanyang96@mail.dlut.edu.cn (D.Z.); 15253803987@163.com (H.J.); 2 Dalian Institute of Chemical Physics, Chinese Academy of Sciences, Dalian 116023, China; lix2013@dicp.ac.cn

**Keywords:** α-glucosidase, MGAM, baicalein, acarbose, synergistic inhibition

## Abstract

**Background**: Combining natural products with conventional antidiabetic agents to inhibit α-glucosidase activity is an effective strategy for preventing postprandial hyperglycemia. Baicalein, a natural flavonoid with well-documented low toxicity, showed potential synergistic effect with acarbose in diabetic models; however, the synergistic performance and mechanisms of the two agents targeting human maltase–glucoamylase (MGAM) remain unclear. **Methods**: Recombinant human MGAM-C and MGAM-N were expressed in *Pichia pastoris* for in vitro inhibition assays. Maltose-loaded mice were used to assess the in vivo hypoglycemic activity and intestinal maltase inhibition. Inhibitor–enzyme interactions were investigated by fluorescence spectroscopy, circular dichroism (CD), multiple molecular docking, and molecular dynamics (MD) simulations. **Results**: Baicalein potently inhibited MGAM-C and MGAM-N with IC_50_ values of 20.41 ± 4.80 μM and 14.04 ± 0.94 μM, respectively, and demonstrated a synergistic effect when combined with acarbose. In vivo, co-administration significantly reduced blood glucose levels and suppressed small intestinal maltase activity in maltose-loaded mice. Mechanistic studies revealed that baicalein functions as a non-competitive inhibitor by binding to the allosteric site of MGAM-C via stable hydrogen bonds with residues Ile1716 and Trp1749. This interaction induces conformational changes in the enzyme’s secondary structure and optimizes the hydrophobic microenvironment of the active site, thereby enhancing the binding affinity and hydrogen bond stability of acarbose. These molecular events collectively contribute to the synergistic inhibition of MGAM-C hydrolytic activity. **Conclusions**: This research revealed the synergistic inhibitory effect of baicalein and acarbose on MGAM and the underlying mechanisms, thereby providing a theoretical basis for developing pharmaceutical formulations to enhance acarbose efficacy.

## 1. Introduction

Diabetes is a significant public health concern. In 2021, it was estimated that 537 million people suffered from diabetes worldwide [1]. Hyperglycemia is a defining characteristic of diabetes [2]. Inhibiting the activities of α-amylase and α-glucosidase to delay the hydrolysis of dietary carbohydrates is an effective strategy for preventing postprandial hyperglycemia [3]. Representative drugs include acarbose, voglibose, and miglitol. Nevertheless, clinical experience with acarbose has revealed that it prolongs the colonic residence of undigested carbohydrates, promoting microbial fermentation and excessive gas production, which often results in unpleasant side effects [4]. Hence, the development of natural product-derived α-glucosidase inhibitors that are both highly potent and safe has attracted considerable research interest.

Baicalein (5,6,7-trihydroxyflavone), a major bioactive flavonoid aglycone, is naturally derived from the dried roots of *Scutellaria baicalensis Georgi* (*Lamiaceae*). As the deglycosylated metabolite of baicalin, it has been shown to exert a broad spectrum of pharmacological properties in cellular and animal models, including antibacterial, antiviral, anti-inflammatory, antioxidant, antipyretic, analgesic, hepatoprotective, and neuroprotective activities, as well as beneficial effects on cardiovascular and neuronal function [5,6,7]. Baicalein has been reported to exhibit an LD50 exceeding 4000 mg/kg in mice, with a subacute no-observed-adverse-effect level (NOAEL) of 2000 mg/kg [8], indicating low toxicity and wide safety window. Emerging evidence further supports its therapeutic potential in the prevention and management of diabetes and associated complications. Our previous study demonstrated that the combination of baicalein and acarbose synergistically inhibited murine α-glucosidase and reduced the risk of progression from prediabetes to type 2 diabetes mellitus (T2DM) in mice by 83.3% [9,10]. Moreover, this combination exhibited a distinct inhibitory effect on starch hydrolysis compared with acarbose alone. However, the underlying mechanism by which baicalein potentiates acarbose-mediated inhibition of α-glucosidase remains unclear. To date, the vast majority of studies have relied on commercially available yeast-derived α-glucosidase (EC: 3.2.1.20) to evaluate its inhibitory activity [11]. Notably, this enzyme differs structurally and catalytically from mammalian α-glucosidases, which may lead to significantly varying results for the same inhibitor when using enzymes from different sources. Unlike the relatively simple structure of yeast α-glucosidase, mammalian α-glucosidase, including maltase–glucoamylase (MGAM) and sucrase–isomaltase (SI), is distributed along the small intestinal brush border membrane [12]. MGAM comprises two catalytic domains, the N-terminal membrane-proximal domain (MGAM-N) and the C-terminal lumen domain (MGAM-C), which are anchored to the small intestine brush border membrane by O-glycosylated linkers derived from the N-terminal domain. MGAM primarily catalyzes the hydrolysis of the α-1,4-glucosidic bond in maltose and small linear chains of glucose (amylose) [13]. With high catalytic activity, it serves as a key enzyme in the hydrolysis of starchy foods.

In this study, the MGAM-C and MGAM-N subunits of human α-glucosidase were successfully expressed as recombinant proteins via the *Pichia pastoris* expression system. Subsequently, the inhibitory effects of baicalein alone and in combination with acarbose, on the human enzymes were evaluated, the mechanism of their synergistic action was investigated, and their hypoglycemic effects were validated in mice. These findings provide insights into the synergistic inhibition of mammalian α-glucosidase by baicalein and acarbose.

## 2. Results and Discussion

### 2.1. Individual and Synergistic Inhibition of Mouse α-Glucosidase by Baicalein and Acarbose

**Figure 1:**
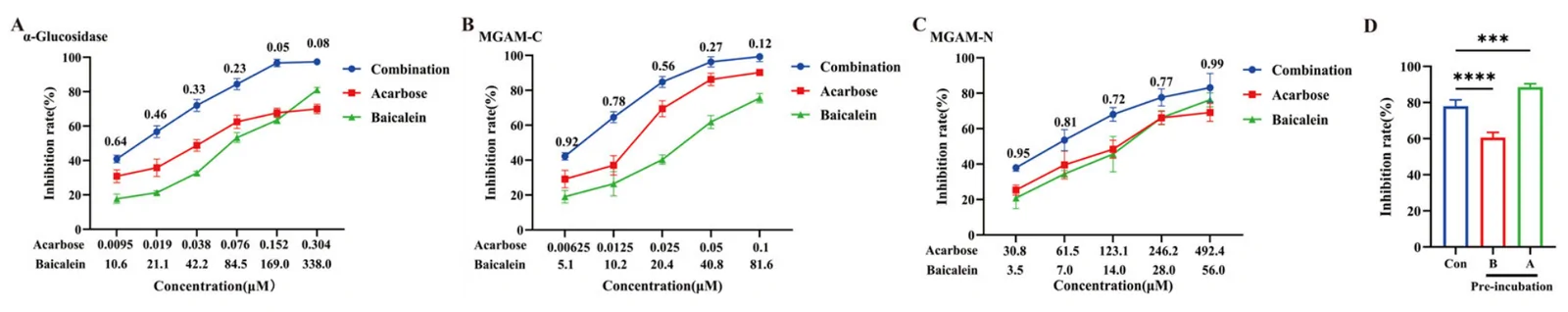
Baicalein and acarbose co-inhibit the activity of mouse α-glucosidase (**A**), MGAM-C (**B**) and MGAM-N (**C**). CI values above data points were calculated by CompuSyn 1.0 software. CI < 0.9, CI = 0.9–1.1, and CI > 1.1 indicate synergism, additive effect, and antagonism, respectively. (**D**) MGAM-C inhibitory activity following combined treatment with baicalein and acarbose under distinct pre-incubation orders. Groups are designated as Con (baicalein and acarbose were pre-incubated together with the enzyme for 30 min prior to substrate addition), B (baicalein pre-incubated for 30 min prior to acarbose addition), and A (acarbose pre-incubated for 30 min prior to baicalein addition). Data are shown as mean ± SEM. **** *p* < 0.0001, *** *p* < 0.001.

The inhibitory activities of baicalein and acarbose on mouse α-glucosidase were initially assayed, with maltose serving as the substrate for the hydrolysis of α-1,4-glycosidic bonds catalyzed by MGAM. Given that α-glucosidase is anchored to the luminal surface of the small intestinal brush border, that the oral absorption rate of acarbose is only approximately 1–2% [14,15], and that baicalein is metabolized in the cecum, only the parent compounds of these two agents were evaluated. The IC_50_ values of baicalein and acarbose on mouse α-glucosidase were 84.52 ± 12.95 μM and 0.076 ± 0.015 μM, respectively (Table 1), which are consistent with previous reports [10,16]. The combined inhibitory effects of baicalein and acarbose were assessed at different ratios of their respective IC_50_ values. Stronger inhibition was observed for the combination than for either compound alone, with combination index (CI) values ranging from 0.05 to 0.64 (Figure 1A), indicating a synergistic interaction. Furthermore, the synergy was found to be more pronounced at higher concentrations, in line with earlier findings [3].

### 2.2. Individual and Synergistic Inhibition of Recombinant MGAM-C/N by Baicalein and Acarbose

Recombinant human MGAM-C and MGAM-N, which are responsible for maltose hydrolysis, were constructed according to a previously reported method [13]. Following a two-step purification procedure, MGAM-C (2.0 mg) and MGAM-N (2.3 mg) were obtained with >99% purity (Figure S1). Functional validation confirmed substantial maltase activity for both isoforms, and the inhibitory activities of baicalein and acarbose against these two enzymes were determined. Acarbose potently inhibited MGAM-C (IC_50_ = 0.025 ± 0.002 μM), but showed markedly weaker activity against MGAM-N (IC_50_ = 123.05 ± 4.45 μM) (Table 1). In contrast, baicalein showed similar inhibitory potency toward both subunits (IC_50_ = 20.41 ± 4.80 μM for MGAM-C; 14.04 ± 0.94 μM for MGAM-N; Table 1), whereas acarbose exhibited a marked subunit preference. Synergy analysis revealed distinct patterns for the two subunits. Combination index (CI) analysis further demonstrated that synergistic inhibition (CI < 0.9) was consistently observed across most concentration combinations for MGAM-C (Figure 1B), with CI values declining to 0.12 at higher concentrations, consistent with the strong synergy previously documented for the inhibition of endogenous mouse small intestinal α-glucosidase (Section 2.1). For MGAM-N, however, the majority of CI values exceeded 0.9, indicating an additive effect; only at intermediate concentrations was weak synergy observed, which diminished at higher concentrations and reverted to additivity (Figure 1C). These results suggest that the synergistic effect of baicalein and acarbose on α-glucosidase is primarily mediated via the MGAM-C subunit.

Our prior work showed that baicalein synergizes with 1-deoxynojirimycin (1-DNJ) against both MGAM-C and MGAM-N [17]. Integrating those findings with the present results suggests a mechanistic principle: synergistic effects are observed only when both components of the combination exhibit strong inhibitory activity against their respective subunits.

To investigate the effect of the addition order of baicalein and acarbose on the combined inhibitory activity, three pre-incubation protocols were designed. As shown in Figure 1D, when baicalein and acarbose were pre-incubated together with the enzyme for 30 min (Con group), the inhibition rate of the combined system against MGAM-C was 77.93%. When baicalein was pre-incubated with the enzyme for 30 min prior to the addition of acarbose and substrate (B group), the inhibition rate was 60.59%. In contrast, when acarbose was pre-incubated with the enzyme for 30 min prior to the addition of baicalein and substrate (A group), the inhibition rate reached 88.55%, representing a 13.63% increase over the Con group. Notably, a marked order-dependent difference was observed between groups B and A: pre-incubation of acarbose with the enzyme prior to baicalein addition (A group) yielded the highest inhibitory activity, whereas pre-incubation of baicalein first (B group) resulted in a relatively lower effect.

This discrepancy suggests that the inhibitory site of baicalein may differ from the active pocket of the enzyme; if both inhibitors targeted the same site, the pre-incubation order would not be expected to produce such a pronounced difference. Based on these observations, it was hypothesized that baicalein binds to a site outside the active pocket and indirectly influences the enzyme hydrolytic activity. From a therapeutic standpoint, the optimal inhibition in the A group (acarbose pre-incubation followed by baicalein) suggests that oral administration of acarbose prior to baicalein may be more effective in controlling postprandial glucose than co-administration or baicalein first. This order advantage also implies that, under the premise of equivalent efficacy, the acarbose dose might be lowered to reduce gastrointestinal adverse effects, offering experimental evidence for optimizing the combined regimen. To verify this, enzyme kinetic assays were conducted to determine the inhibition type of baicalein against MGAM-C.

### 2.3. Inhibition Kinetics of Baicalein and Acarbose

**Figure 2:**
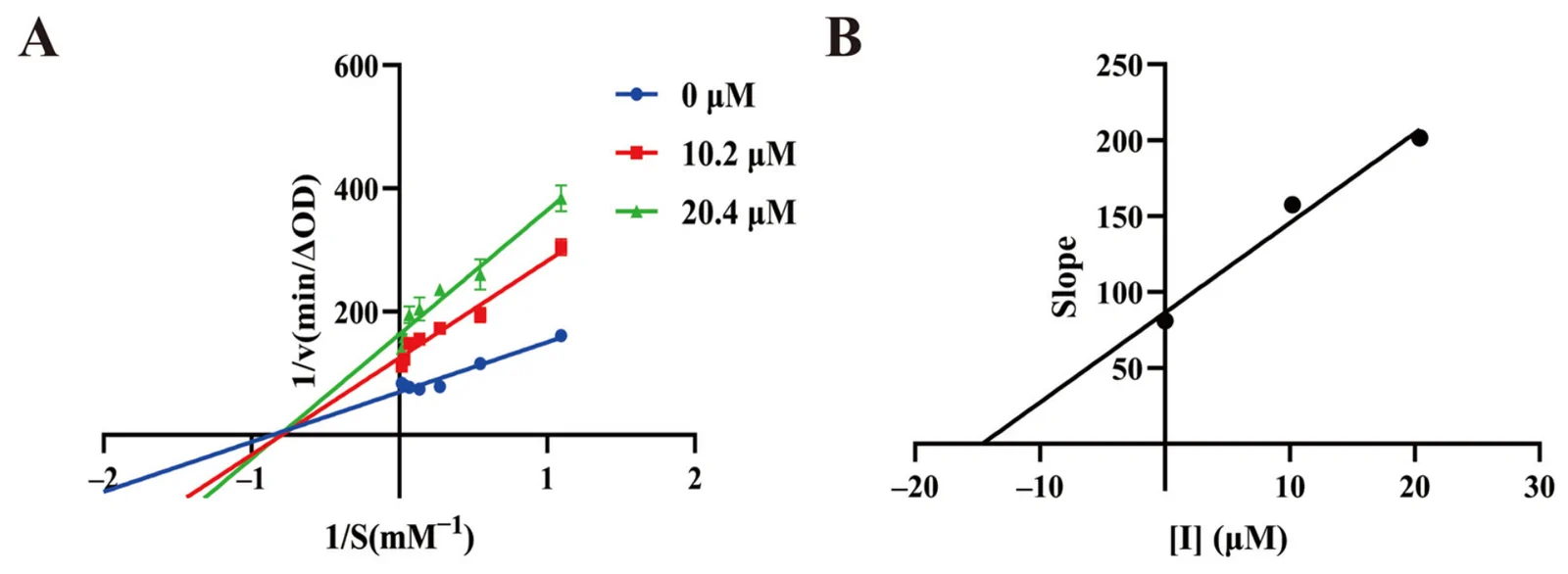
Lineweaver−Burk plots of MGAM-C against with baicalein (**A**). The secondary plot represents slope versus [*I*] (**B**).

The inhibition mechanism of baicalein against MGAM-C was investigated using Lineweaver–Burk analysis (Figure 2A). With increasing baicalein concentrations, the lines intersected at a common point on the negative x-intercept, a characteristic pattern of non-competitive inhibition. In accordance with this kinetic profile, *V_max_* was markedly reduced relative to the control, whereas *K_m_* remained statistically invariant, confirming that baicalein binds to an allosteric site distinct from the catalytic center (Table S2). The corresponding *K_i_* value was determined to be 14.59 μM. In addition, the slope exhibited a strong linear dependence on baicalein concentration, suggesting a single binding site for baicalein on MGAM-C (Figure 2B). Acarbose, in contrast, is a competitive inhibitor that binds to the active site of α-glucosidase [18]. The mutually exclusive binding modes, with acarbose at the catalytic pocket and baicalein at an allosteric site, provide a structural rationale for their simultaneous occupancy of MGAM-C and thus underpin the observed synergistic enzyme inhibition [19].

### 2.4. Fluorescence Quenching Analysis

The intrinsic fluorescence of α-glucosidases, arising from aromatic amino acids residues (Trp, Phe, Tyr) upon excitation at 280 nm or 295 nm, is quenched by the addition of aromatic compounds. This quenching, resulting from π-π interactions, is widely used to study protein-ligand binding [20,21]. Fluorescence spectra of MGAM-C were recorded in the presence of acarbose alone, baicalein alone, or both compounds together.

**Figure 3:**
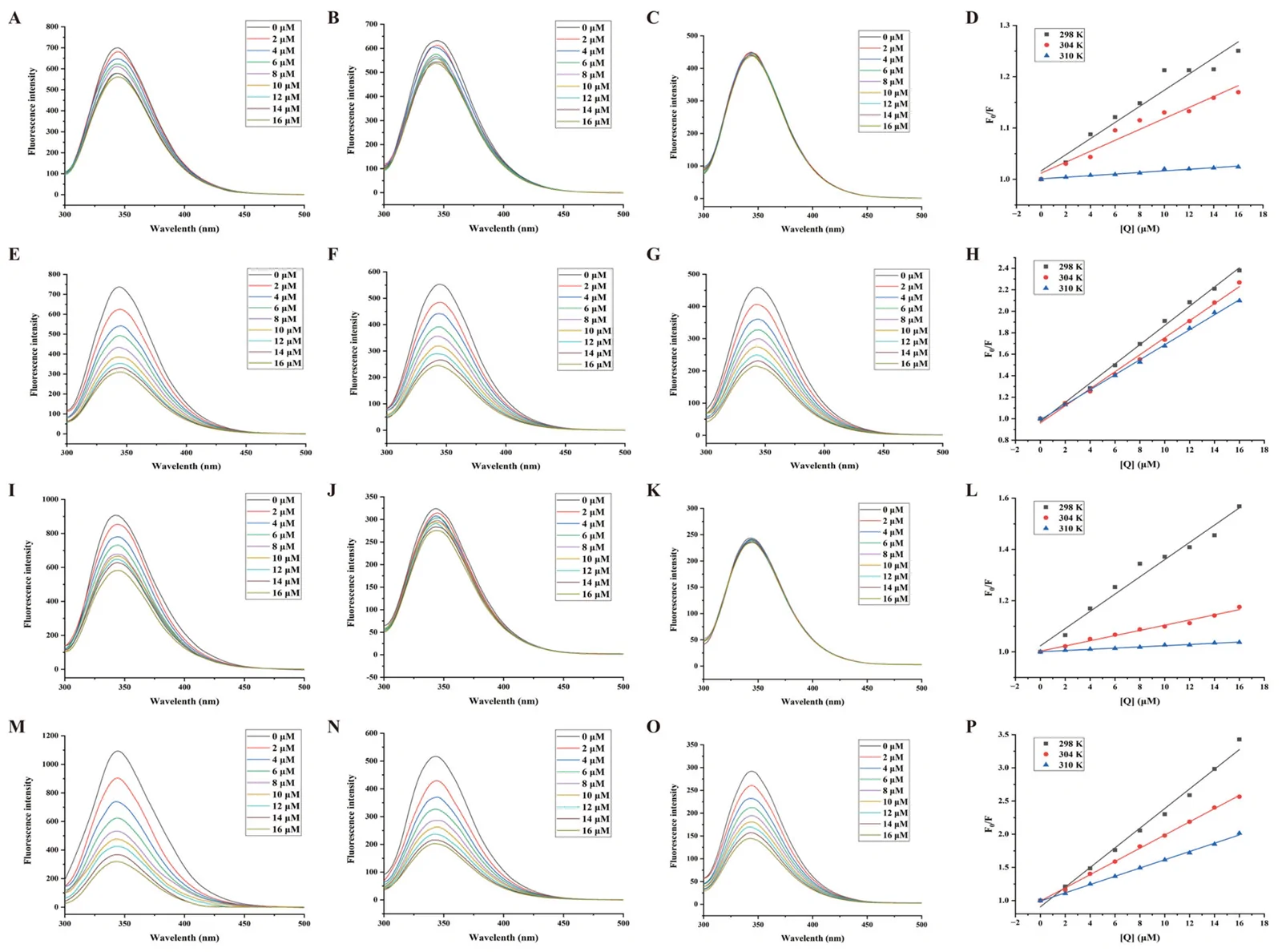
Stern–Volmer analysis of MGAM-C fluorescence quenching by acarbose and baicalein. (**A**–**C**) Quenching spectra of MGAM-C with acarbose at 298–310 K; (**D**) Stern–Volmer plots. (**E**–**G**) Quenching spectra of MGAM-C with baicalein at 298–310 K; (**H**) Stern–Volmer plots. (**I**–**K**) Quenching spectra of MGAM-C with acarbose in the presence of 4 μM baicalein (298–310 K); (**L**) Stern–Volmer plots. (**M**–**O**) Quenching spectra of MGAM-C with baicalein in the presence of 4 μM acarbose (298–310 K); (**P**) Stern–Volmer plots.

As illustrated in Figure 3A–C,E–G, the intrinsic fluorescence intensity of MGAM-C was progressively quenched upon incremental addition of acarbose or baicalein, indicating effective quenching via direct molecular interaction. Stern–Volmer analysis showed that the quenching constants (*K_SV_*) for acarbose and baicalein decreased with rising temperature (Table 2), a trend characteristic of static quenching. The linear Stern–Volmer plots, with decreasing slopes at 298, 304, and 310 K (Figure 3D,H) indicated a single dominant quenching mechanism for each inhibitor binding to MGAM-C. Moreover, the calculated bimolecular quenching rate constants (*K_q_*) for both compounds significantly exceeded the diffusion-controlled limit (2.0 × 10^10^ L·mol^−1^·s^−1^), further supporting static quenching mediated by ground-state complex formation between MGAM-C and each inhibitor. The double-logarithmic plots of acarbose and baicalein for MGAM-C, calculated using Equation (8), exhibited strong linear correlations (Figure S5) [22,23,24]. The values of *K_a_* and n were determined from the intercept and slope of the plots, respectively, as shown in Table 2. The observed decrease in *K_a_* with rising temperature suggested that complex formation is reversible. Notably, the derived n values for both MGAM-C_Acarbose and MGAM-C_Baicalein systems were approximately unity (*n* ≈ 1.0), indicating a single, well-defined binding site for each inhibitor on MGAM-C.

Hydrophobic interactions, van der Waals forces, electrostatic interactions, and hydrogen bonds constitute the four principal non-covalent forces that govern protein–ligand binding [25]. Thermodynamic parameters for the reaction were calculated using Equation (9). As shown in the table, the negative Gibbs free energy changes (Δ*G* < 0) observed across all tested temperatures confirmed that the binding of both acarbose and baicalein to MGAM-C is thermodynamically spontaneous. For acarbose, Δ*H* = −205.99 kJ·mol^−1^ and Δ*S* = −613.93 J·mol^−1^·K^−1^; for baicalein, Δ*H* = −84.84 kJ·mol^−1^ and Δ*S* = −178.65 J·mol^−1^·K^−1^. The negative values of both Δ*H* and Δ*S* indicate that the binding events are predominantly enthalpy-driven and are stabilized by van der Waals interactions and hydrogen bonds.

To investigate the synergistic mechanism between acarbose and baicalein, MGAM-C solutions were pre-incubated with either baicalein or acarbose. Upon subsequent addition of acarbose, the fluorescence intensity of the MGAM-C_Baicalein complex gradually decreased. The *K_a_* values of acarbose with the complex increased by 196%, 38%, and 100% at 298 K, 304 K, and 310 K, respectively, indicating that baicalein effectively enhances the binding affinity of acarbose to MGAM-C. Conversely, when baicalein was added to the MGAM-C_Acarbose complex, progressive fluorescence quenching was also observed, and its *K_a_* increased by 94%, 49%, and 14% at the corresponding temperatures relative to binding to free MGAM-C, suggesting reciprocal positive cooperativity between the two inhibitors. Similar results were observed in our previous experiments combining baicalein with 1-DNJ [17]. Furthermore, the n values for both MGAM-C_Baicalein_Acarbose and MGAM-C_Acarbose_Baicalein binding were approximately equal to 1, indicating that acarbose and baicalein still bind to single, non-overlapping sites. Enzyme kinetic analyses revealed competitive inhibition by acarbose and non-competitive inhibition by baicalein. Fluorescence spectroscopic data further revealed the molecular basis of the synergistic interaction between baicalein and acarbose. Baicalein binds to the non-competitive site of MGAM-C, while acarbose occupies the active site. When both inhibitors coexisted in the reaction system, the binding constant (*K_a_*) of each inhibitor was significantly increased compared to that in the single-inhibitor condition, irrespective of the order of addition. This finding indicates that the binding of the two inhibitors to the enzyme exhibits a mutually reinforcing positive cooperative effect, which underlies the synergistic potentiation of inhibitory activity observed in the combination system in vitro.

### 2.5. Synchronous Fluorescence Spectroscopy

**Figure 4:**
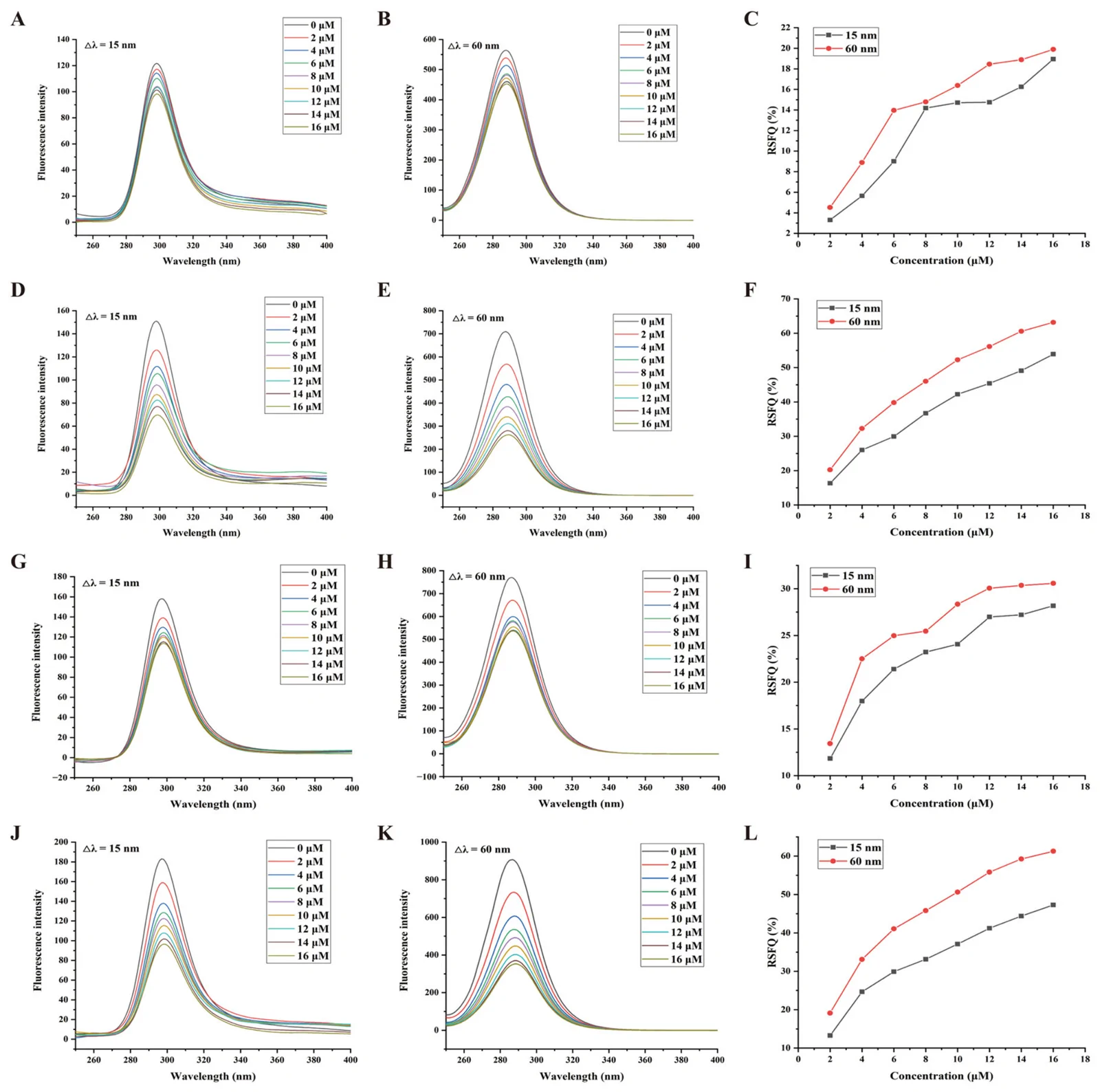
Synchronous fluorescence spectra and RSFQ values for MGAM-C with acarbose and baicalein. MGAM-C with acarbose at Δλ = 15 nm (**A**) and Δλ = 60 nm (**B**), and RSFQ values (**C**). MGAM-C with baicalein at Δλ = 15 nm (**D**) and Δλ = 60 nm (**E**), and RSFQ values (**F**). MGAM-C with acarbose in the presence of 4 μM baicalein at Δλ = 15 nm (**G**) and Δλ = 60 nm (**H**), and RSFQ values (**I**). MGAM-C with baicalein in the presence of 4 μM acarbose at Δλ = 15 nm (**J**) and Δλ = 60 nm (**K**), and RSFQ values (**L**).

Synchronous fluorescence spectroscopy at fixed wavelength intervals of Δλ = 15 nm and Δλ = 60 nm was used to probe the microenvironmental changes in tyrosine (Tyr) and tryptophan (Trp) residues in α-glucosidase, respectively [19]. As shown in Figure 4, the fluorescence intensities of Tyr and Trp gradually decreased with increasing concentrations of acarbose and baicalein, indicating that both compounds interact with the Tyr and Trp residues of MGAM-C. Under single-inhibitor conditions, the quenching at Δλ = 60 nm consistently exceeded that at Δλ = 15 nm, and a red shift of approximately 1 nm was detected exclusively in the Trp synchronous spectrum, suggesting that both inhibitors induce greater polarity and a modest reduction in local hydrophobicity around Trp residues. In the combination system, the presence of baicalein increased the relative synchronous fluorescence quenching (RSFQ) values of acarbose from 18.95% to 28.18% at Δλ = 15 nm and from 19.89% to 30.58% at Δλ = 60 nm, suggesting that baicalein enhanced the binding affinity of acarbose for both Tyr and Trp residues via an indirect mechanism. Conversely, acarbose reduced the RSFQ values of baicalein from 53.90% to 47.27% at Δλ = 15 nm and from 63.17% to 61.26% at Δλ = 60 nm, suggesting that acarbose might partially attenuate the interaction of baicalein with these residues.

### 2.6. CD Spectra

**Figure 5:**
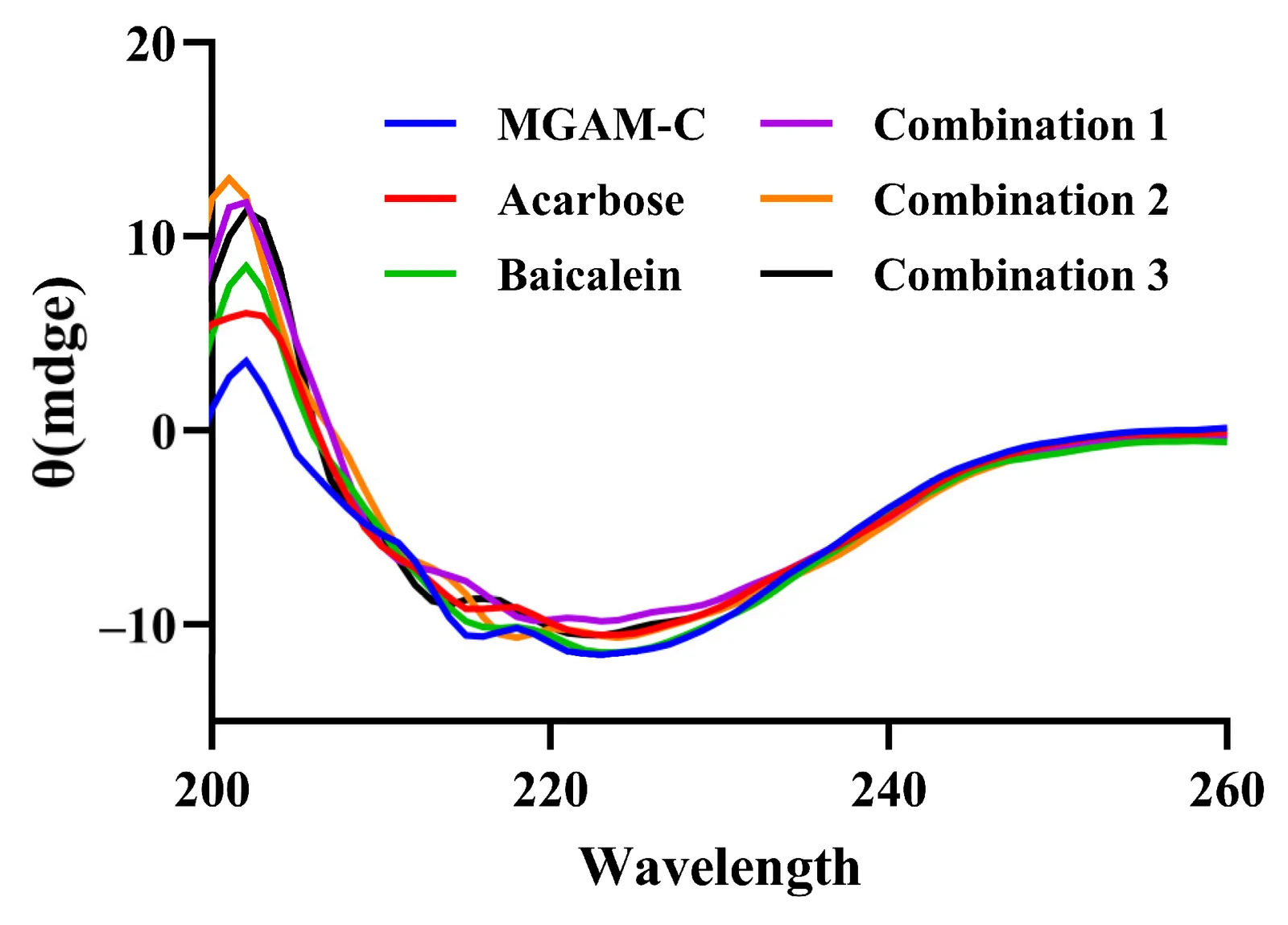
CD spectra of MGAM-C in the presence of acarbose and/or baicalein. Combination 1: Simultaneous addition of acarbose and baicalein to MGAM-C; Combination 2: Pre-incubation of MGAM-C with baicalein, followed by titration with acarbose; Combination 3: Pre-incubation of MGAM-C with acarbose, followed by titration with baicalein.

Ligand binding can induce structural changes in proteins, which is critical for their mechanism of action and biological function. Circular dichroism (CD) spectroscopy is capable of revealing the distinct structural features of chiral molecules in different spectral regions [3]. This technique was therefore employed to monitor secondary structure changes in MGAM-C upon addition of baicalein and acarbose. As shown in Figure 5, the CD spectra exhibited two negative CD absorption bands near 209 nm and 220 nm, characteristic of α-helical structures [19]. In the presence of baicalein and acarbose, the CD intensity of these α-helical bands decreased, accompanied by a slight peak shift, indicating that the inhibitors interacted with MGAM-C and induced conformational changes in the protein.

Individual addition of acarbose or baicalein induced a slight increase in α-helix content, accompanied by an increase in β-sheet content (from 34.36% to 37.08% and 36.04%, respectively) and decreases in β-turn (from 18.27% to 16.96% and 17.13%) and random coil (from 37.44% to 35.90% and 36.83%) (Table S3), reflecting enhanced structural order of the protein. Co-administration of both inhibitors resulted in more pronounced conformational changes, with a greater increase in α-helix and more marked reductions in β-turn and random coil contents compared with single inhibitors. Furthermore, pre-incubation of MGAM-C with baicalein before acarbose addition led to the most significant alterations in α-helix and random coil contents, suggesting that baicalein promoted the formation of a more stable secondary structure upon subsequent acarbose binding.

### 2.7. Molecular Docking

**Figure 6:**
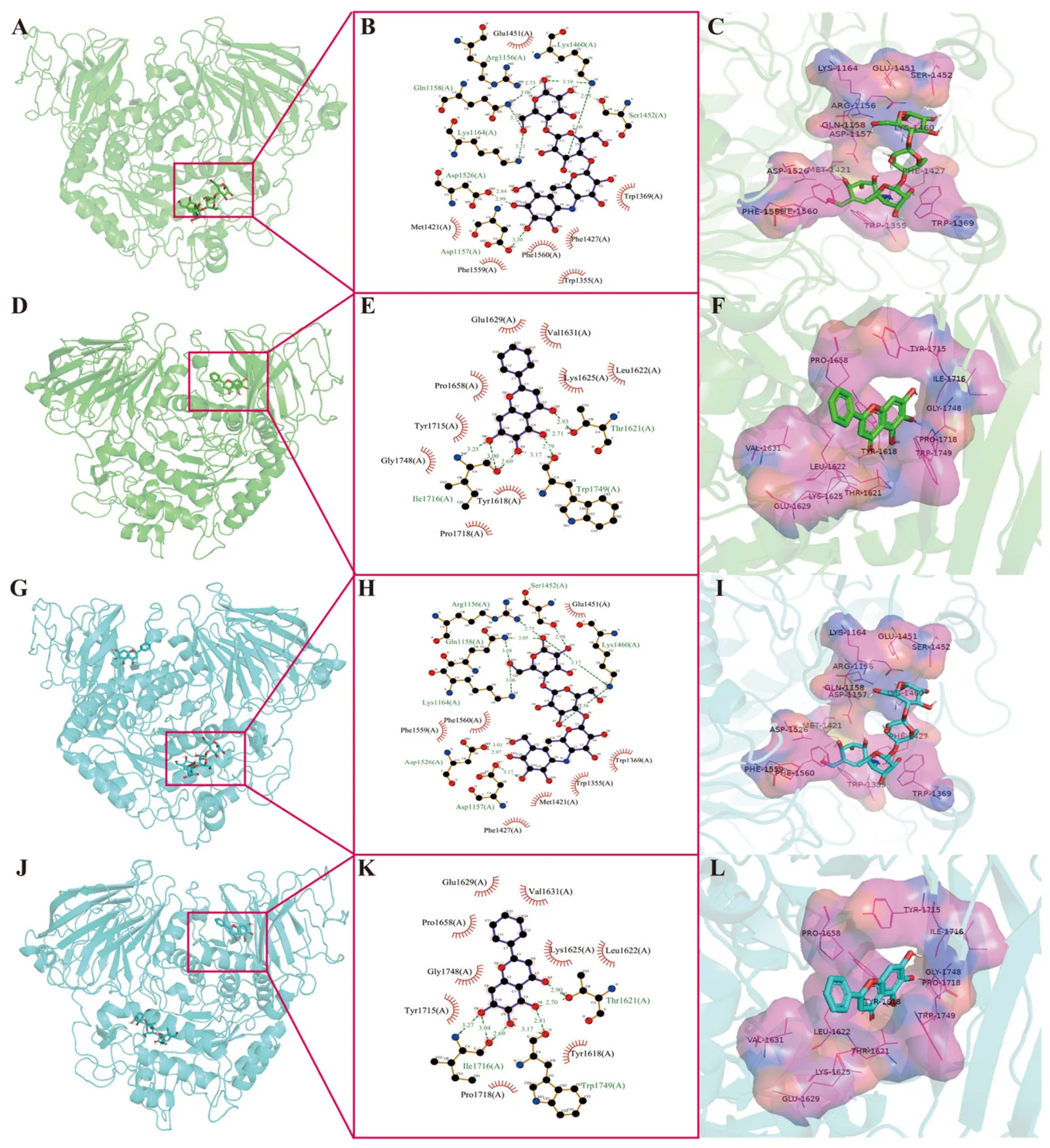
The docking results and interaction between inhibitors and 3TON. (**A**,**D**) are the binding sites of acarbose and baicalein on 3TON, respectively, (**B**,**E**) are the 2D diagrams of their interaction, and (**C**,**F**) are their binding pockets. (**G**) is the binding site of acarbose on 3TON_Baicalein complex, (**H**) is 2D diagram of its interaction, and (**I**) is its binding pocket. (**J**) is the binding site of baicalein on 3TON_Acarbose complex, (**K**) is 2D diagram of its interaction, and (**L**) is its binding pocket.

To identify the structural determinants of inhibitor binding to MGAM-C, molecular docking simulations were performed using AutoDock Vina 1.2.0 against the crystal structure of human MGAM-C (PDB ID: 3TON). Acarbose was docked to the active site of 3TON with a binding energy of −4.6 kcal/mol (Figure 6A) [13]. The molecule is embedded in a hydrophobic pocket formed by residues Pro1159, Trp1355, Trp1369, Met1421, Phe1427, Glu1451, Phe1559, and Phe1560 and forms 10 hydrogen bonds with Arg1156, Asp1157, Gln1158, Ser1452, Lys1460, Arg1510, and Asp1526 (Figure 6B, Table S4). Baicalein bound to the same allosteric site distant from the active site as previously reported [17], with a binding energy of −9.3 kcal/mol, consistent with its non-competitive inhibitory profile. Owing to its compact molecular scaffold, baicalein penetrates deeply into this allosteric pocket (Figure 6F) and engages in hydrophobic interactions with Tyr1618, Leu1622, Lys1625, Gln1629, Val1631, Pro1658, Tyr1715, Pro1718, and Gly1748 (Figure 6E). Additionally, specific hydrogen bonds are formed: Thr1621 with the 5-OH of ring A and the C4 carbonyl of ring C; Ile1716 with the 6-OH (one bond) and 7-OH (two bonds) of ring A; and Trp1749 with both the 5-OH and 6-OH of ring A. These interactions highlight the critical role of hydroxyl groups in mediating the inhibitory activity of baicalein [26].

To explore the mechanism underlying the synergistic effect of baicalein and acarbose, the binding properties of the single inhibitor and the 3TON-inhibitor complex were investigated. Compared with acarbose alone, the binding energy of acarbose at the active site decreased from −4.6 to −4.8 kcal/mol in the presence of baicalein. Although the interacting amino acid residues remained unchanged (Figure 6H,I), the lengths of seven hydrogen bonds were shortened (Table S4), indicating that the presence of baicalein stabilizes the complex by strengthening local interactions [27]. In the 3TON_Acarbose_Baicalein complex, the binding energy of baicalein at the allosteric site slightly decreased from −9.3 to −9.4 kcal/mol compared with baicalein alone. Although the binding site remained unchanged, the lengths of three hydrogen bonds increased, resulting in a marginal decrease in stability. Collectively, these findings demonstrate that the two compounds mutually enhance each other’s binding affinity to MGAM-C, consistent with the fluorescence spectroscopy results. While previous docking studies have largely focused on single inhibitors interacting with α-glucosidase, simultaneous docking of multiple ligands remains underexplored. In this study, this approach was extended by docking acarbose and baicalein within the MGAM-C complex, offering structural insights into their synergistic inhibition. However, given the limitations of the rigid docking approach in reproducing the binding poses of highly flexible ligands, molecular dynamics simulations were further performed to validate the stability of the predicted binding mode.

### 2.8. Binding Stability

**Figure 7:**
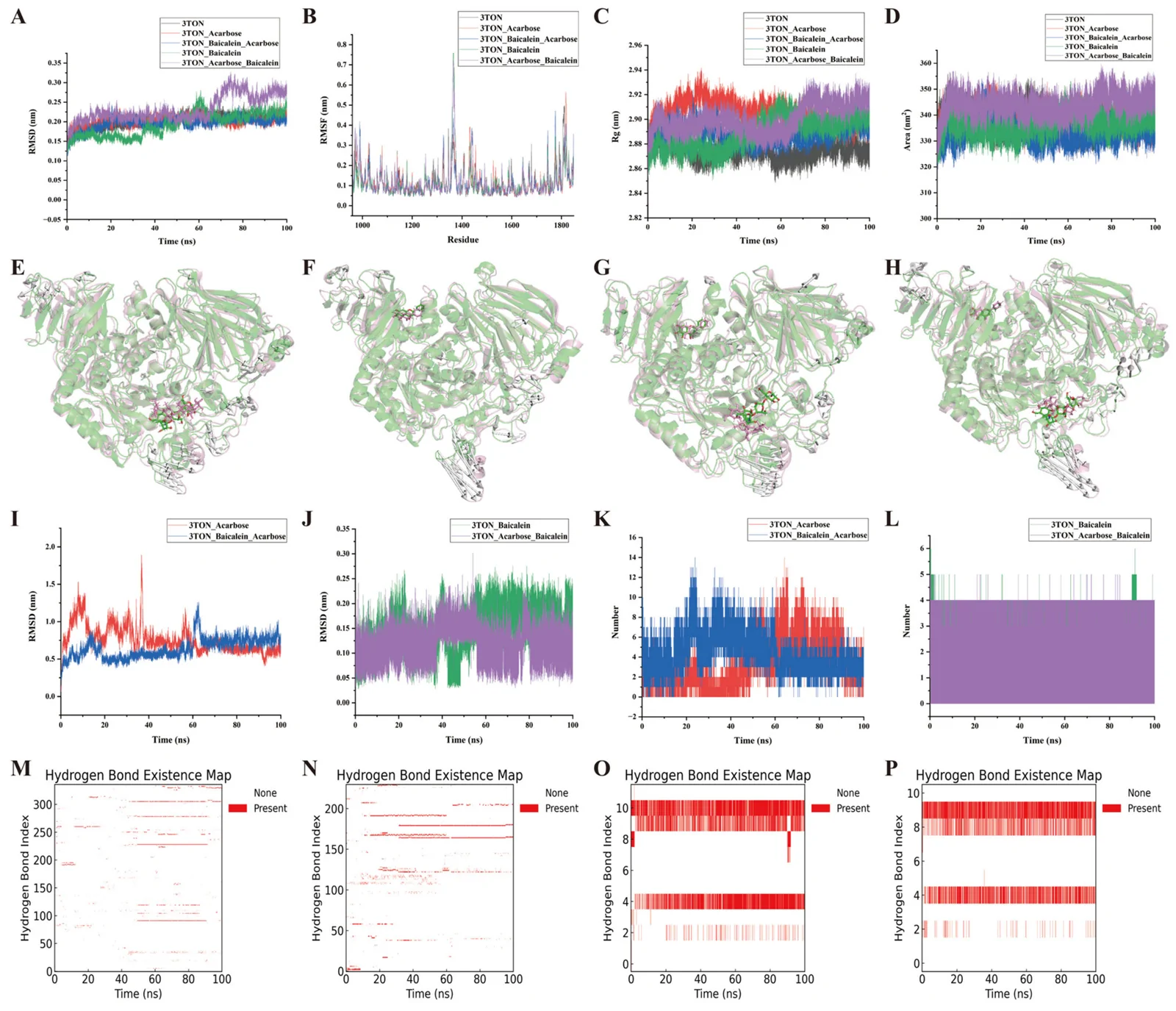
MD simulation results. (**A**) RMSD; (**B**) RMSF; (**C**) Rg; (**D**) SASA. The direction of motion of the 3TON main chain during molecular dynamics simulations: (**E**) 3TON_Acarbose, (**F**) 3TON_Baicalein, (**G**) 3TON_Baicalein_Acarbose, and (**H**) 3TON_Acarbose_Baicalein; (**I**) RMSD for acarbose; (**J**) RMSD for baicalein; (**K**) hydrogen bonding of acarbose; (**L**) hydrogen bonding of baicalein; (**M**) the hydrogen bond occupancy of acarbose in 3TON_Acarbose; (**N**) the hydrogen bond occupancy of acarbose in 3TON_Baicalein_Acarbose; (**O**) the hydrogen bond occupancy of baicalein in 3TON_Baicalein; (**P**) the hydrogen bond occupancy of baicalein in 3TON_Acarbose_Baicalein.

Molecular dynamics (MD) simulation is a powerful approach for the real-time assessment of protein-ligand interactions [28]. Based on the docking results, five complex systems were constructed: 3TON, 3TON_Acarbose, 3TON_Baicalein_Acarbose, 3TON_Baicalein, and 3TON_Acarbose_Baicalein, where the order of ligand names corresponds to the sequence of ligand introduction. Protein stability and conformational variation upon inhibitor binding were evaluated by root mean square deviation (RMSD) [29]. As shown in Figure 7A, the 3TON, 3TON_Acarbose, and 3TON_Baicalein_Acarbose systems reached equilibrium approximately 20 ns after the start of the simulation. The 3TON_Baicalein system exhibited a gradual increase in RMSD after 35 ns, with notable perturbation near 60 ns, before stabilizing at approximately 0.2202 nm. In the 3TON_Acarbose_Baicalein system, the RMSD values rose sharply and fluctuated considerably after 60 ns, reaching levels substantially higher than those of all other systems. These observations indicate that while pre-binding of baicalein enhances the stability of the subsequent acarbose-bound system, adding baicalein to an already stabilized acarbose complex unexpectedly disrupts its structural integrity.

RMSF is a key metric for assessing protein flexibility during MD simulations, reflecting local fluctuations of individual amino acid residues [30]. As shown in Figure 7B, during the 100 ns simulation of free 3TON, the N- and C-terminal residues along with Gly1365, Ser1366, and Trp1369 showed RMSF fluctuations exceeding 0.3 nm. Upon ligand binding, the 1360–1369 region, which comprises peripheral random coils and α-helices, showed the most pronounced fluctuations, with RMSF values predominantly above 0.3 nm. Notably, in the 3TON_Baicalein and 3TON_Acarbose_Baicalein systems, the RMSF of residues 1364–1366 exceeded 0.6 nm, indicating that baicalein markedly increased the flexibility of the protein’s peripheral regions.

The radius of gyration (Rg) reflects the change in the overall structural compactness of the protein throughout the MD simulation [31]. Throughout the simulation, all ligand-bound systems exhibited higher Rg values than free 3TON (~2.8735 nm), suggesting reduced compactness upon ligand binding (Figure 7C). The highest Rg value (2.9097 nm) was observed for the 3TON_Acarbose_Baicalein system upon further addition of baicalein, indicating the most expanded spatial conformation and the lowest molecular integrity. In contrast, a lower Rg (2.8901 nm) was observed for the 3TON_Baicalein_Acarbose system than for the other complexes, reflecting greater compactness.

Solvent-accessible surface area (SASA) reflects the contact surface area between solvent molecules and protein complexes, serving as an indicator of protein–solvent interactions [3]. As shown in Figure 7D, addition of baicalein induced a more loosely packed and unstable conformation, accompanied by enhanced protein–solvent interactions and weakened intra-protein contacts. Addition of acarbose alone slightly increased the SASA value; however, pre-binding with baicalein resulted in a lower SASA value for the 3TON_Baicalein_Acarbose system with subsequent acarbose addition compared with free 3TON, suggesting that baicalein suppressed acarbose-induced solvent exposure and promoted a more compact protein conformation.

A visual analysis of the backbone motion directions in the complex system during the molecular dynamics simulation is presented (Figure 7E–H and Figure S6). The addition of acarbose induced an ordered counterclockwise motion in the peripheral loop regions of 3TON, while simultaneously stabilizing the loops near the allosteric site. With baicalein pre-bound, the loop regions adjacent to the allosteric site exhibited only minor perturbations throughout the simulation. The overall structure of the 3TON_Baicalein_Acarbose complex demonstrated greater compactness and structural stability, which is conducive to sustained and robust binding of acarbose at the active site. When added alone, baicalein suppressed localized loop disorder at its binding site but notably induced pronounced conformational unfolding near the active site. The 3TON_Acarbose_Baicalein system exhibited significantly increased loop mobility around both the active and allosteric sites, accompanied by overall structural loosening, reduced conformational stability, and increased solvent exposure.

### 2.9. Ligand RMSD and Hydrogen Bond Analysis

The RMSD fluctuations of the ligand molecules were analyzed throughout the simulation. The behavior of acarbose in the 3TON_Acarbose and 3TON_Baicalein_Acarbose systems was compared, as well as that of baicalein in the 3TON_Baicalein and 3TON_Acarbose_Baicalein systems. As shown in Figure 7I, in the presence of acarbose alone, the acarbose molecule exhibited an unstable conformation within the active site during the initial 0–60 ns. After 60 ns, the molecule gradually approached equilibrium and fluctuated around an RMSD of 0.6386 nm, with a slight decrease observed near 92 ns. In the 3TON_Baicalein_Acarbose system, the RMSD of acarbose remained consistently low during the first 60 ns, but showed a notable fluctuation near 60 ns and then attained a new equilibrium state at 0.7706 nm. Pre-binding of baicalein thus contributed substantially to maintaining the conformational stability of acarbose. The RMSD values of baicalein in the single-agent and combination systems were 0.1746 nm and 0.1094 nm, respectively, indicating that baicalein was more stable when both inhibitors were present during the simulation.

Intermolecular hydrogen bonds are critical for the stability of inhibitor–enzyme complexes. Although molecular docking revealed multiple hydrogen bonds between acarbose or baicalein and 3TON, the majority of these hydrogen bonds were not stabilized during the simulations (Figure 7M–P). As shown in Figure 7K, acarbose alone formed an average of 5.13 hydrogen bonds, yet none achieved stable occupancy (>50%) (Table S5), a commonly used threshold for defining stable hydrogen bonds [32]. In the presence of baicalein, the hydrogen-bonding occupancy of acarbose increased (Figure 7N), with O11 and O13 of acarbose forming relatively stable hydrogen bonds with Asp1526, exhibiting occupancies of 60.203% and 62.819%, respectively. This suggests that baicalein binding at the allosteric site enhances the stability of acarbose’s hydrogen bonding at the active site. Compared to acarbose, the 5-, 6-, and 7-hydroxyl groups of baicalein penetrated more deeply into the allosteric pocket (Figure S7B,D), forming stable hydrogen bonds with surrounding residues. However, the interaction between baicalein and Trp was weakened in the presence of acarbose. The occupancies of the two hydrogen bonds formed between Trp1749 and baicalein decreased from 79.722% and 61.639% to 67.062% and 46.912%, respectively, consistent with the synchronous fluorescence spectroscopy results.

### 2.10. Binding Energy Analysis

**Figure 8:**
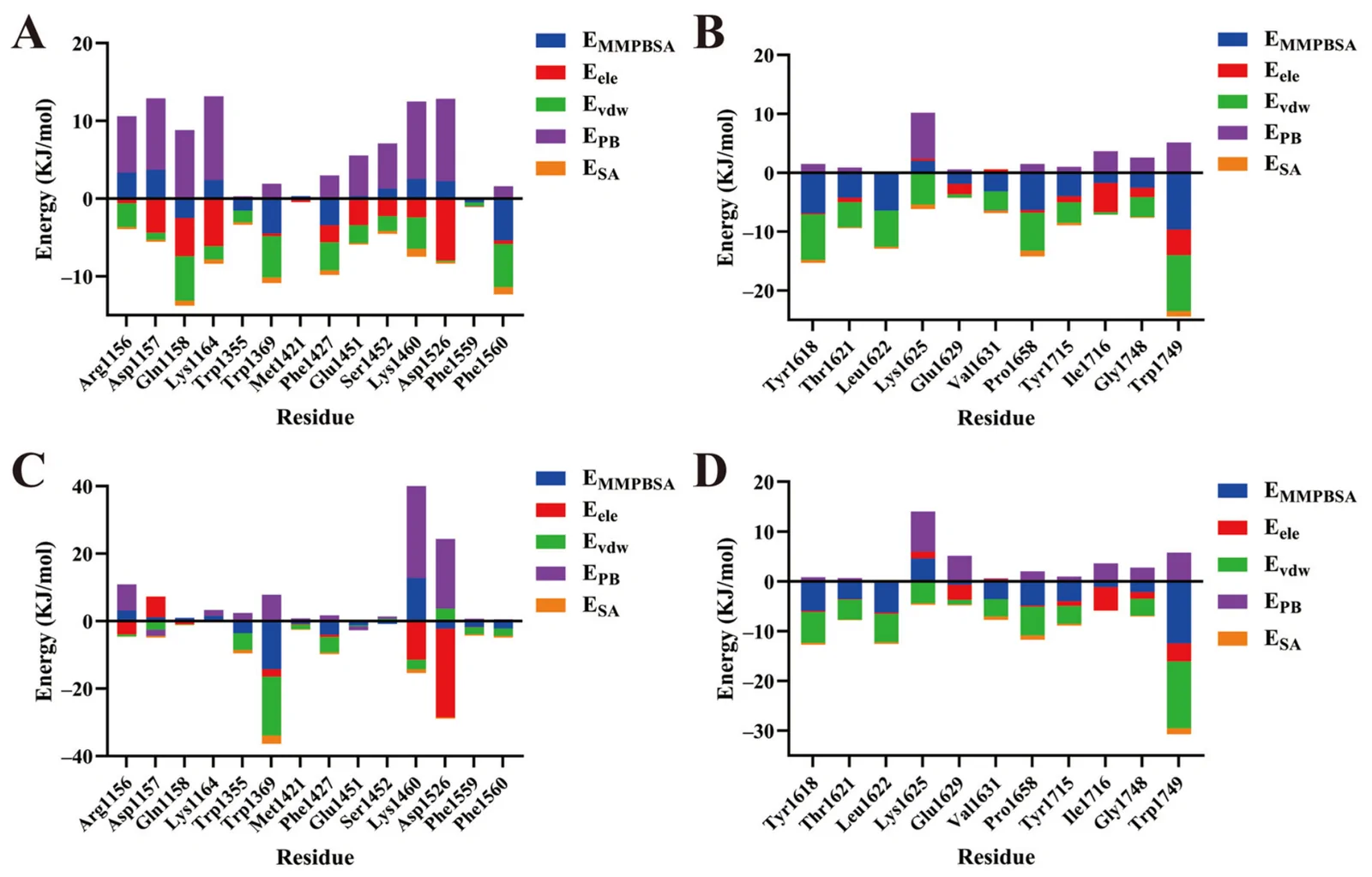
The contribution of per residue of MGAM-C to the binding free energy. (**A**) Key amino acids at the active site of 3TON_Acabose. (**B**) Key amino acids at the allosteric site of 3TON_Baicalein. (**C**) Key amino acids at the active site of 3TON_Baicalein_Acabose. (**D**) Key amino acids at the allosteric site of 3TON_Acarbose_Baicalein.

By decomposing the energy contributions of individual residues, the amino acids critical for binding interactions were systematically analyzed (Figure 8). Among the four energy components, electrostatic interactions (E_ele_), van der Waals forces (E_vdw_), and nonpolar solvation energy (E_SA_) all contributed favorably to complex stability. In contrast, the polar solvation energy (E_PB_) was positive and unfavorable for binding, likely because the protein binding pocket is typically a hydrophobic environment, and the complex tends to stabilize via nonpolar interactions during molecular dynamics (MD) simulations. When acarbose was used alone, most residues in the binding pocket exhibited high E_PB_ values. However, in the presence of baicalein, acarbose was able to bind within a more hydrophobic and enclosed pocket (Figure S7A,C), leading to reduced E_PB_ values for these residues. Notably, the van der Waals contribution of Trp1369 and the electrostatic contribution of Asp1526 were significantly enhanced, promoting acarbose binding in the active pocket. For baicalein, pre-binding of acarbose to the active site enhanced its van der Waals interactions with Trp1749 but weakened its electrostatic interactions, which may account for the reduced stability of its hydrogen bonds.

In the 3TON_Acarbose_Baicalein system, the active site is first occupied by acarbose, inducing a compact conformation but at the cost of sharply elevated solvent polarization energy (E_PB_) at the binding site. Baicalein binds to the allosteric pocket when added subsequently; however, effective allosteric communication to the catalytic center is not elicited by this binding, because the active site is already occupied. Consequently, steric and conformational incompatibility is generated between the two ligand-binding sites, driving the protein into a globally relaxed conformation. The binding free energy is dominated by unfavorable polar solvation energy, indicating failed entropy-enthalpy compensation [33]. Although distinct regions are stabilized by each ligand individually, their opposing effects lead to destabilization of the complex [34]. The native conformation is disrupted by this conflict, disordering catalytic residues such as Asp1526 in the active pocket and abolishing the precise three-dimensional conformation required for hydrolytic activity. As enzymatic activity strictly depends on conformational integrity [35], structural destabilization directly results in functional inactivation, thereby contributing to the synergistic inhibition by acarbose and baicalein.

In the 3TON_Baicalein_Acarbose complex, baicalein first occupies the allosteric site and forms stable hydrogen bonds with Ile1716 and Trp1749, altering the active-site gate conformation through residue interactions [36]. This binding induces a hydrophobic, low-polarity microenvironment in the active pocket, thereby reducing the polar solvation penalty for subsequent acarbose binding. Consequently, O11 and O13 of acarbose are enabled to form stable, high-occupancy hydrogen bonds with Asp1526, while the van der Waals contribution of Trp1369 is enhanced, rendering the entire complex more compact and stable. Thus, pre-binding of baicalein to the allosteric site optimizes the active-site conformation, providing acarbose with a thermodynamically favorable hydrophobic closed pocket that promotes its stable binding and competitive inhibition. These findings support the hypothesis, derived from enzymatic assays, that baicalein binds to an allosteric site of MGAM-C. Nevertheless, definitive identification of the precise allosteric binding site and the specific amino acid residues involved in baicalein interaction will require further investigations, such as co-crystallization of MGAM-C with baicalein, or site-directed mutagenesis coupled with ITC/SPR binding assays.

In summary, molecular dynamics simulations revealed that the synergistic enhancement of inhibitory activity was observed irrespective of ligand addition order—whether acarbose was introduced prior to baicalein or vice versa. However, the underlying mechanistic pathways differed: pre-binding of acarbose induced conformational instability in the enzyme, whereas pre-binding of baicalein modulated the conformation and dynamics of the active site. These observations are consistent with prior reports demonstrating that ligand binding sequences lead to different mechanisms of action [37]. In line with the experimental findings on the order of inhibitor addition, the superior inhibitory effect observed when acarbose was added first suggests that conformational destabilization of the enzyme plays a more critical role in its functional regulation. Collectively, these findings provide further theoretical support for understanding the molecular basis of combination therapy involving natural products and clinical drugs.

### 2.11. Postprandial Glycemic Response Measurement

**Figure 9:**
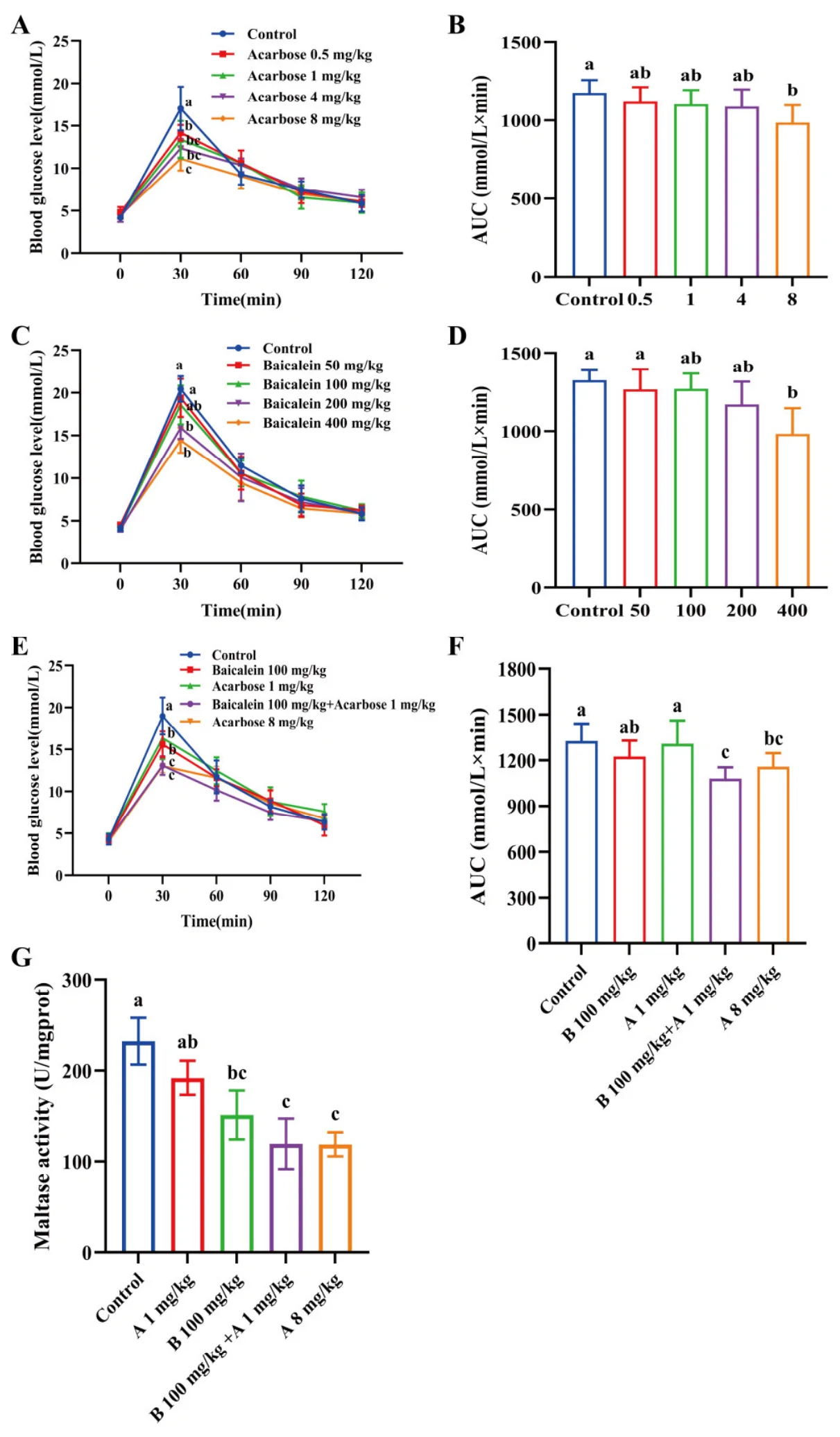
PBG-lowering effect of acarbose or baicalein administered alone or in combination in maltose tolerance test. (**A**,**C**,**E**) show dynamic blood glucose profiles after maltose load in C57BL/6 mice orally administered with acarbose and/or baicalein. (**B**,**D**) represent the AUC for acarbose and baicalein alone, respectively. (**F**) represent the AUC for the combined treatment at two different concentrations. (**G**) represents the activity levels of brush border maltase in the small intestine. Data were analyzed by ANOVA and post hoc Dunnett’s test. Means with different letters differ significantly in each sub-figure (*p* < 0.05).

To evaluate the hypoglycemic effects of the combination of baicalein and acarbose in mammals, oral maltose tolerance tests were performed in mice, and blood glucose levels were measured at 0, 30, 60, 90, and 120 min after maltose loading (2 g/kg) (Figure 9). Postprandial blood glucose (PBG) levels in the control group peaked at 30 min and subsequently decreased. Both baicalein and acarbose, when administered alone, reduced PBG levels in a dose-dependent manner compared with the control group. Administration of 200 mg/kg and 400 mg/kg baicalein alone significantly decreased PBG levels at 30 min. The 400 mg/kg dose also significantly reduced the area under the curve (AUC). Acarbose alone at 0.5, 1, 4, and 8 mg/kg significantly decreased PBG levels at 30 min, but not at 60 min, indicating that acarbose lowers the blood glucose level by delaying the postprandial glycemic rise. Only 8 mg/kg of acarbose significantly reduced AUC, indicating that 8 mg/kg is the minimum dose to reduce AUC following 2 g/kg maltose load. In contrast, when acarbose (1 mg/kg) was combined with baicalein (100 mg/kg), both the 30 min PBG and AUC were significantly lowered (*p* < 0.05, Figure 9E,F). Moreover, the high-dose combination showed no significant difference from acarbose monotherapy at 8 mg/kg, highlighting its potential as a combined therapeutic strategy.

Based on the maltose tolerance test results, which identified the maximal suppression of postprandial blood glucose in mice at 30 min post-administration, this time point was selected for terminal tissue collection. Jejunal mucosal tissues were harvested to quantify maltase activity, thereby directly evaluating the local inhibitory effect of test compounds on intestinal carbohydrate hydrolysis. As shown in Figure 9G, co-administration of baicalein (100 mg/kg) and acarbose (1 mg/kg) significantly suppressed jejunal mucosal maltase activity relative to the control group following a maltose load. Notably, the magnitude of inhibition achieved by this combination showed no significant difference compared with that elicited by high-dose acarbose individually (8 mg/kg; *p* > 0.05). This result indicates that the combination of baicalein and acarbose synergistically inhibits jejunal maltase activity, enabling acarbose to achieve an enzyme inhibitory effect comparable to that of a high-dose acarbose alone at just one-eighth of the equivalent dose. This provides direct biochemical evidence supporting the synergistic pharmacodynamic interaction between baicalein and acarbose in the intestinal lumen.

Our previous study demonstrated that the combination of acarbose and baicalein significantly reduces postprandial blood glucose levels in mice subjected to a sucrose loading test [10], suggesting a potential inhibitory effect on the SI-C subunit. In the present study, a recombinant human MGAM subunit was employed in in vitro assays, and the hypoglycemic potential of this combination regimen was further confirmed under both in vivo and in vitro conditions. Collectively, these findings demonstrate that the co-administration of baicalein and acarbose effectively delays maltose hydrolysis in the small intestine and prevents postprandial hyperglycemia. This work provides new insights for the design and development of potential drugs targeting maltose hydrolysis and offers a theoretical basis for developing dietary supplements to be used alongside acarbose in treatment regimens.

The NOAEL of Baicalein has been reported as high as 2000 mg/kg [8], while the highest oral dose of baicalein in the present study was 400 mg/kg, and only 100 mg/kg was used in the combination group, both well below the NOAEL threshold, suggesting favorable safety at these dose levels. Acarbose was administered at a maximum dose of 8 mg/kg, also far below the previously reported dose range associated with toxic effects [38]. The animal experimental results demonstrated that the combination of the two inhibitors produced superior hypoglycemic effects compared with monotherapy even at relatively low doses, implying that combined administration may further broaden the safety window by enabling dose reduction. Pharmacokinetically, acarbose was reported to be predominantly excreted in faeces as the intact drug, with a low oral absorption rate, and the intestinal metabolism of baicalein was previously characterized by our group [39]; moreover, no significant interaction or structural alteration between the two compounds was observed in vitro, providing a preliminary basis for predicting the pharmacokinetic behavior of the combination. Furthermore, although the present MGAM inhibition study was specifically focused on the starch hydrolysis step, it was found in our previous study in prediabetic mouse models that 8-week administration of this combination ameliorated insulin resistance and non-alcoholic fatty liver disease through the inhibition of de novo lipogenesis [40], suggesting that the combination may exert long-term benefits via additional mechanisms. Nevertheless, given the differences between the combination and its individual components, the safety, pharmacokinetics, and long-term efficacy of the combination need further evaluation in future studies. In this study, the effect of the administration order of baicalein and acarbose on their combined inhibitory activity was also evaluated using an in vivo maltose tolerance test, in which acarbose was administered by gavage first, followed by baicalein 30 min later. However, this experimental approach did not yield conclusive results, primarily because the repeated gavage within a short time interval elicited substantial stress responses, leading to marked variability in blood glucose measurements and consequently compromised the statistical analysis of glycemic levels. Therefore, future investigations employing approaches that induce minimal blood glucose fluctuation are warranted to rigorously assess how the dosing order influences the in vivo synergistic efficacy of these two compounds.

## 3. Materials and Methods

### 3.1. Materials and Reagents

Acarbose was purchased from Bayer Healthcare Co., Ltd. (Berlin, Germany). All flavonoid standard products were purchased from Sichuan Victory Biological Technology Co., Ltd. (Chengdu, China). Maltose was purchased from Beijing Solarbio Science & Technology Co., Ltd. (Beijing, China). The glucose test kit (ACCU-CHEK) used for in vivo assays was purchased from Roche Diagnostics GmbH (Mannheim, Germany). The glucose assay kit for in vitro assay was purchased from Shanghai Rongsheng Biopharmaceutical Co., Ltd. (Shanghai, China). The maltase assay kit was purchased from the Nanjing Jiancheng Institute of Biological Engineering (Nanjing, China). All other reagents and solvents were of analytical grade.

### 3.2. MGAM Preparation and Maltase Inhibition Activity Assay

The recombinant MGAM-C (amino acids 87–954) and MGAM-N (amino acids 960–1853) were constructed in our laboratory using a previously described method with modifications [13]. Both proteins were expressed in *Pichia pastoris* GS115 using the pPIC9K vector, induced with 1% methanol, and secreted into BMMY medium. The proteins were harvested 3–5 days post-induction and purified by Ni Sepharose affinity chromatography followed by Sephadex G-200 gel filtration. SDS-PAGE confirmed a purity of 99% for both proteins, with expected molecular weights of approximately 110 kDa.

Mammalian crude α-glucosidase was prepared from mouse small intestine as previously described [10]. Intestinal tissue was homogenized in ice-cold PBS (100 mM, pH 7.4) and centrifuged at 12,000× *g* for 15 min; the supernatant was collected as the enzyme source.

α-Glucosidase activity toward maltose was assayed using a modified method [17]. Acarbose was dissolved in phosphate buffer, and baicalein was dissolved in 50% DMSO. Enzyme solution (0.4 U/mL, 50 μL) was pre-incubated with test compounds at 37 °C for 30 min, followed by addition of maltose (1% *w*/*v* final concentration) and incubation at 37 °C for 20 min. The reaction was terminated by heating at 100 °C for 5 min. Glucose released was quantified using the glucose oxidase method. Control experiments confirmed that baicalein, at the concentrations tested, did not interfere with the glucose oxidase assay (Figure S2). The final DMSO concentration (10%) had no effect on enzyme activity. IC_50_ values were calculated using GraphPad Prism 10.4.

### 3.3. Synergistic Inhibition Activity

Baicalein and acarbose were added to the reaction system at concentrations corresponding to multiples of their IC_50_ values, and the inhibition rates for both single and combined use were determined. The synergistic index (CI) of baicalein and acarbose was calculated by Compusyn software 1.0 [41]. This quantification is based on the median-effect principle developed by Chou and Talalay, which measures the extent of inhibitor interaction. The equation for the median-effect principle is as follows:

(1) $$\log\left(\frac{f_{a}}{f_{u}}\right)\;=\;m\log D\;-\;m\log D_{m}\;$$

where *f*_a_ is the fraction affected by dose *D*, *f*_u_ is the unaffected fraction (*f*_u_ = 1 − *f*_a_), *m* is the coefficient signifying the shape of the dose–effect curve, *D* is the dose of the inhibitor, and *D*_m_ is the median-effect dose (IC_50_ in this article).

The equation for the CI is expressed as follows:

(2) $$\mathrm{CI}\;=\frac{{\left(D\right)}_{1}}{{\left(D_{X}\right)}_{1}}+\frac{{\left(D\right)}_{2}}{{\left(D_{X}\right)}_{2}}$$

where (*D*)_1_ and (*D*)_2_ represent the defined concentrations of inhibitor 1 and inhibitor 2, respectively, at which the combination of the two inhibitors produces a specified rate of inhibition. (*D*_x_)_1_ and (*D*_x_)_2_ denote the concentrations at which each inhibitor alone achieves this same rate of inhibition. According to the CI values, the combined inhibition was categorized as follows: synergistic (CI < 0.9), additive (CI = 0.9–1.1), or antagonistic (CI > 1.1). The standardized isobolograms are shown in Figure S4.

### 3.4. Determination of the Inhibitory Activity of the Combined MGAM-C System Under Different Pre-Incubation Orders of the Two Components

To ensure sufficient binding between the inhibitors and the enzyme and to allow the reaction to reach equilibrium, a 30 min pre-incubation period was adopted as the standard procedure for determining the α-glucosidase inhibitory activity of flavonoids. The working concentrations of baicalein and acarbose were fixed at their respective IC_50_ values. To investigate the effect of pre-incubation order on the combined inhibitory activity, the following three experimental groups were designed: (1) baicalein and acarbose were pre-incubated together with the enzyme for 30 min, followed by the addition of substrate to initiate the reaction (Con group); (2) baicalein was pre-incubated with the enzyme for 30 min, followed by the simultaneous addition of acarbose and substrate to initiate the reaction (B group); and (3) acarbose was pre-incubated with the enzyme for 30 min, followed by the simultaneous addition of baicalein and substrate to initiate the reaction (A group). According to the MGAM-C enzyme activity assay system described in Section 2.2, all components were added sequentially. After completion of the pre-incubation step, the enzymatic reaction was initiated and the inhibition rate of each system was measured.

### 3.5. Kinetic Study of Enzymatic Inhibition

The inhibition kinetics of baicalein and acarbose were investigated using the same procedure in the activity assay. To analyze the competitive inhibition mechanism, the Lineweaver–Burk equation [42] can be expressed in double reciprocal form as follows:

(3) $$\frac{1}{v}\;=\;\frac{K_{m}}{V_{max}}\left(1+\frac{\left[I\right]}{K_{i}}\right)\frac{1}{\left[S\right]}+\frac{1}{V_{max}}$$

To analyze the noncompetitive inhibition mechanism, the Lineweaver–Burk equation [19] can be expressed in double reciprocal form as follows:

(4) $$\frac{1}{v}\;=\;\frac{K_{m}}{V_{max}}\left(1+\frac{\left[I\right]}{K_{i}}\right)\frac{1}{\left[S\right]}+\frac{1}{V_{max}}(1+\frac{\left[I\right]}{\alpha K_{i}})$$

Secondary plots were constructed as follows:

(5) $$\mathrm{slope}\;=\;\frac{K_{m}}{V_{max}}+\frac{K_{m}[I]}{V_{max}K_{i}}$$

To analyze the mixed inhibition mechanism, the Lineweaver–Burk equation [42] can be expressed in double reciprocal form as follows:

(6) $$\frac{1}{v}\;=\;\frac{K_{m}}{V_{max}}\left(1+\frac{\left[I\right]}{K_{i}}\right)\frac{1}{\left[S\right]}+\frac{1}{V_{max}}(1+\frac{\left[I\right]}{\alpha {K_{i}}^{\prime }})$$

where v is the rate of the enzymatic reaction in the absence and presence of the inhibitor, *K_i_* is the inhibition constant, *K_m_* is the Michaelis–Menten constant, [*I*] is the inhibitor concentration, [*S*] is the substrate concentration, and α is the apparent coefficient denoting the ratio of the noncompetitive inhibition constant to competitive inhibition constant. The slope of the secondary plot was also determined. The corresponding enzyme kinetic parameters, including *K_m_* and *V_max_*, were further determined by nonlinear regression analysis.

### 3.6. Fluorescence Spectra Analysis

Fluorescence spectra were recorded on a fluorescence spectrometer (F7100) with slight modifications to a described method [43]. The excitation wavelength was set at 280 nm, and emission spectra were collected from 300 to 500 nm at three temperatures (298, 304, and 310 K), with slit widths of 10 nm. MGAM-C (0.05 mg/mL, 2.5 mL) in a 1.0 cm quartz cuvette was pre-incubated and then continuously titrated with baicalein or acarbose. The mixture was equilibrated for 10 min prior to each measurement. All spectra were baseline-corrected by subtracting the buffer signal.

The Stern–Volmer equation was employed to determine the fluorescence quenching constant Ksv:

(7) $$\frac{F_{0}}{F}\;=\;1\;+\;K_{q}\tau_{0}[Q]\;=\;1\;+\;K_{SV}[Q]$$

*F*_0_ denotes the fluorescence intensity in the absence of inhibitor; *K_SV_* represents the Stern–Volmer quenching constant; [*Q*] indicates the concentration of the inhibitor; *K_q_* and *τ*_0_ denote the fluorescence quenching constant and the lifetime of the fluorophore, respectively.

Use the following equation to calculate the binding constant K_a_ and the number of binding sites *n*:

(8) $$\log\frac{F_{0}-F}{F}\;=\;\log K_{a}\;+\;n\log[Q]$$

The definitions of *F*_0_, *F*, and [*Q*] are consistent with those in Equation (7).

The thermodynamic parameters associated with intermolecular interactions were investigated using the van’t Hoff equation. The calculation is expressed as follows:

(9) $$\log K_{a}\;=-\frac{△H}{RT}+\frac{△S}{R}$$

Δ*H*, Δ*S*, and Δ*G* denote the changes in enthalpy, entropy, and Gibbs free energy, respectively, and R represents the gas constant (8.314 J·mol^−1^·K^−1^).

The synchronous fluorescence spectra of the samples were acquired by setting the wavelength intervals between excitation and emission (Δ*λ* = *λem* − *λex*) to 15 nm and 60 nm, respectively. The synchronous fluorescence quenching ratio (RSFQ) was calculated according to the following equation:

(10) $$\mathrm{RSFQ}\;=\;1\;-\;\frac{F}{F_{0}}$$

The three-dimensional fluorescence spectra of the samples were also recorded. The excitation (EX) spectra were acquired over a range of 200 to 350 nm, and the emission (EM) spectra were recorded from 250 to 500 nm, with a step interval of 10 nm. All other experimental conditions were maintained consistently with those used in the fluorescence spectroscopy measurements.

### 3.7. Determination of Circular Dichroism (CD)

Far-UV CD spectra (200–250 nm) of MGAM-C were recorded on a Bio-Logic MOS 500 spectrometer (BioLogic Science Instruments, Claix, France). The concentration of MGAM-C was maintained at 2.0 × 10^−5^ mol/L, while the concentrations of baicalein and acarbose were 5.0 × 10^−5^ and 5 × 10^−6^ mol/L, respectively. Spectra were scanned at 100 nm/min with a 1 nm bandwidth. All data are expressed as mean residue ellipticity [θ] (mdeg). The actual CD spectrum of the enzyme was obtained by subtracting the solvent spectrum acquired under identical conditions in the presence of the inhibitor alone. Secondary structure content was calculated by CDNN.

### 3.8. Molecular Docking Assay

Molecular docking was performed using AutoDock Vina 1.2.0 to predict the binding mode between MGAM-C and inhibitors. The crystal structure of MGAM-C (PDB: 3TON) was obtained from the Protein Data Bank (www.rcsb.org, accessed on 15 January 2024). During the docking procedure, all water molecules were removed, hydrogen atoms were added, and Gasteiger charges were assigned. The 3D structures of baicalein (PubChem CID: 5281605) and acarbose (PubChem CID: 9811704) were downloaded from the PubChem database (https://pubchem.ncbi.nlm.nih.gov/, accessed on 15 January 2024), followed by addition of hydrogen atoms and energy minimization. Ligand and receptor files were converted to pdbqt format using AutoDockTools (ADT). The grid box was configured to encompass the entire molecule. Among the generated docking poses, the model with the lowest docking energy was selected as the most favorable conformation. PyMOL 2.4.1 and LigPlot 2.3.1 were used for visualization and analysis.

### 3.9. Molecular Dynamics Simulation

Molecular dynamics (MD) simulations of the initial structures were performed from the molecular docking results, selecting the conformation with the lowest binding energy. MD simulations were performed using GROMACS 2018.8. The AMBER99SB-ILDN force field was applied and the ligand topology was generated using the Acpype server. Each system was placed in a cubic solvent box filled with SPC water molecules and ions were added to neutralize the net charge. Then, the simulations of energy minimization, Constant number of particles, volume and temperature (NVT) (300 K), and Constant number of particles, pressure and temperature (NPT) (1 bar) ensembles were performed to equilibrate the system [44]. A 100 ns MD simulation was performed for the equilibrated system.

### 3.10. Oral Maltose Tolerance Test and Jejunal Maltase Activity Measurement

The oral maltose tolerance test was conducted as previously described [45,46]. C57BL/6 mice (20–23 g, 6 weeks old) were purchased from Liaoning Changsheng Biotechnology Co., Ltd. (Shenyang, China). Mice were housed five per cage under standard conditions (22 °C, 12:12 h light/dark cycle) with free access to standard pellet feed and water for one week to acclimatize to the environment. After one week of acclimatization, mice were fasted for 12 h and randomly assigned to the following groups: control, baicalein (50, 100, 200, and 400 mg/kg), and acarbose (0.5, 1, 4, and 8 mg/kg). Maltose (2 g/kg body weight) was administered by gavage simultaneously with baicalein or acarbose. Blood samples were collected from the tail vein at 0, 30, 60, 90, and 120 min, and blood glucose levels were measured using a glucometer (ACCU-CHEK). The area under the curve (AUC) was calculated using the trapezoidal method.

In addition, separate group of mice was subjected to the maltose tolerance test under the same conditions and euthanized at 30 min post-administration for immediate collection of jejunal tissue. Jejunal maltase activity was measured using a commercial assay kit.

### 3.11. Statistical Analysis

The results were expressed as mean values ± standard deviation (*n* = 3). One-way analysis of variance (ANOVA) was implemented using GraphPad Prism 10.4 followed by multiple tests. Statistical significance was defined as *p* < 0.05.

Sample size was estimated a priori using G*Power software 3.1.9.7 for one-way ANOVA. With five experimental groups, α = 0.05, target power = 0.8, and a large effect size f = 0.5 derived from published carbohydrate tolerance assays with distinct hypoglycemic intergroup differences, the theoretical required sample size was 11 mice per group (55 animals in total). In the present study, 10 mice were assigned to each group (50 mice overall). Post hoc power analysis revealed the actual statistical power reached 0.78, an acceptable level for acute single-bolus carbohydrate tolerance experiments. Consistent with most published short-term oral glucose/maltose tolerance studies [45,47,48].

## 4. Conclusions

In summary, this study comprehensively delineates the molecular mechanism underlying the synergistic inhibition of human MGAM-C, a key catalytic subunit of intestinal α-glucosidase, by baicalein alone and in combination with acarbose. Enzyme kinetic and spectroscopic analyses revealed that baicalein acts as a non-competitive inhibitor by specifically binding to the allosteric site of MGAM-C, thereby modulating protein conformation through a static quenching mechanism primarily driven by van der Waals forces and hydrogen bonds. Circular dichroism spectroscopy further confirmed that this binding event induces structural changes in the enzyme, characterized by increased α-helix and β-sheet contents alongside reduced random coil proportions. Moreover, investigation of the inhibitor addition order revealed that pre-incubation of acarbose with the enzyme yielded superior inhibitory efficacy of the combination, suggesting that the conformational dynamics mediated by the order of addition play a critical role in modulating enzyme function. Multiple molecular docking and molecular dynamics simulations demonstrated that ligand addition order dictates distinct synergistic mechanisms: when acarbose binds prior to baicalein, its occupation of the active site impedes subsequent allosteric communication, leading to conformational conflict and enhanced inhibition via global destabilization; conversely, when baicalein binds first, it occupies the allosteric site and induces pre-organization of the active pocket into a hydrophobic, closed conformation—thereby facilitating stable acarbose binding. Although both sequences yield synergistic inhibition, their underlying mechanistic pathways are fundamentally distinct. In vivo experiments in a maltose-loaded mouse model further corroborated the synergistic effect, showing a marked reduction in blood glucose levels and suppression of jejunal maltase activity. Collectively, these findings provide a mechanistic basis for the development of allosterically enhanced combination therapies targeting α-glucosidase and establish a scientific foundation for designing pharmaceutical formulations aimed at MGAM inhibition.

## Figures and Tables

**Table 1:** IC_50_ values of baicalein and acarbose against mouse α-glucosidase and recombinant MGAM.

	Baicalein (μM)	Acarbose (μM)
α-Glucosidase	84.52 ± 12.95	0.076 ± 0.015
MGAM-C	20.41 ± 4.80	0.025 ± 0.002
MGAM-N	14.04 ± 0.94	123.05 ± 4.45

**Table 2:** Quenching constants (K_SV_), binding constants (Ka), and thermodynamic parameters for the interaction of acarbose and baicalein with MGAM-C at different temperatures.

	T (K)	K_SV_ (×10^4^ L·mol^−1^)	K_q_ (×10^12^ L·mol^−1^·s^−1^)	R	K_a_ (×10^4^ L·mol^−1^)	R	n	∆H (kJ·mol^−1^)	∆S (J·mol^−1^·K^−1^)	∆G (kJ·mol^−1^)
MGAM-C_A	298	1.48 ± 0.16	1.48 ± 0.16	0.9223	0.94 ± 0.04	0.9555	0.94	−205.99	−613.93	−23.04
	304	1.00 ± 0.10	1.00 ± 0.10	0.9296	0.29 ± 0.03	0.9460	0.87			−19.36
	310	0.15 ± 0.01	0.15 ± 0.01	0.9496	0.04 ± 0.01	0.9711	0.87			−15.67
MGAM-C_B	298	9.09 ± 0.20	9.09 ± 0.20	0.9948	36.38 ± 0.12	0.9956	1.13	−84.84	−178.65	−31.60
	304	8.14 ± 0.21	8.14 ± 0.21	0.9951	15.94 ± 0.06	0.9938	1.07			−30.53
	310	7.04 ± 0.11	7.04 ± 0.11	0.9981	9.68 ± 0.10	0.9991	1.03			−29.46
MGAM-C_B_A	298	3.24 ± 0.24	3.24 ± 0.24	0.9622	2.78 ± 0.02	0.9666	0.98	−224.92	−669.59	−25.38
	304	1.00 ± 0.06	1.00 ± 0.06	0.9753	0.40 ± 0.02	0.9848	0.93			−21.36
	310	0.23 ± 0.01	0.23 ± 0.01	0.9826	0.08 ± 0.01	0.9852	0.91			−17.34
MGAM-C_A_B	298	15.31 ± 0.61	15.31 ± 0.61	0.9890	70.69 ± 0.15	0.9982	1.14	−119.16	−288.29	−33.25
	304	9.98 ± 0.16	9.98 ± 0.16	0.9982	23.69 ± 0.09	0.9938	1.08			−31.52
	310	6.27 ± 0.12	6.27 ± 0.12	0.9972	10.99 ± 0.18	0.9970	1.05			−29.79

## Data Availability

The original contributions presented in this study are included in the article. Further inquiries can be directed to the corresponding author.

## Supplementary Materials

The following supporting information can be downloaded at https://www.mdpi.com/article/10.3390/ph19081215/s1: Figure S1: The SDS-PAGE analysis of MGAM-C and MGAM-N proteins. (A) fermentation broth; (B) purified by Ni Sepharose; (C) purified by Sephadex G-200; Figure S2: The impact of baicalein’s antioxidant properties on the determination of glucose concentration using the glucose oxidation method; Figure S3. The inhibitory effects of acarbose and baicalein on mouse α-glucosidase (A,B), MGAM-C (C,D) and MGAM-N (E,F), respectively; Figure S4. Normalized isobologram plots depicting the inhibitory effects of the combination of acarbose and baicalein on enzymes. (A) α-Glucosidase. Point 1: 0.0095 μM Acarbose + 10.5625 μM Baicalein, Point 2: 0.019 μM Acarbose + 21.125 μM Baicalein, Point 3: 0.038 μM Acarbose + 42.25 μM Baicalein, Point 4: 0.076 μM Acarbose + 84.5 μM Baicalein, Point 5: 0.152 μM Acarbose + 169 μM Baicalein, Point 6: 0.304 μM Acarbose + 338 μM Baicalein; (B) MGAM-C. Point 1: 0.00625 μM Acarbose + 5.1 μM Baicalein, Point 2: 0.0125 μM Acarbose + 10.2 μM Baicalein, Point 3: 0.025 μM Acarbose + 20.4 μM Baicalein, Point 4: 0.05 μM Acarbose + 40.8 μM Baicalein, Point 5: 0.1 μM Acarbose + 81.6 μM Baicalein; (C) MGAM-N. Point 1: 30.775 μM Acarbose + 3.5 μM Baicalein, Point 2: 61.55 μM Acarbose + 7 μM Baicalein, Point 3: 123.1 μM Acarbose + 14 μM Baicalein, Point 4: 246.2 μM Acarbose + 28 μM Baicalein, Point 5: 492.4 μM Acarbose + 56 μM Baicalein; Figure S5. Double-logarithmic plot of MGAM-C. (A) MGAM-C_Acarbose; (B) MGAM-C_Baicalein; (C) MGAM-C_Baicalein_Acarbose; (D) MGAM-C_Acarbose_Baicalein; Figure S6. The direction of motion of the free 3TON main chain during molecular dynamics simulations; Figure S7. The binding pockets of the inhibitors on 3TON after MD (The binding pocket comprises atoms within a 4 Å radius around the molecule.). (A) Acarbose on 3TON_Acarbose; (B) Baicalein on 3TON_Baicalein; (C) Acarbose on 3TON_Baicalein_Acarbose complex; (D) Baicalein on 3TON_Acarbose_Baicalein complex; Table S1. Individual inhibition of baicalein and acarbose against mouse α-glucosidase and recombinant MGAM; Table S2. Kinetic parameters for MGAM of baicalein; Table S3. Changes of secondary structure of baicalein, acarbose and combination when acting on MGAM-C; Table S4. Details of molecular docking analysis between inhibitor to 3TON; Table S5. Occupancy percentages of hydrogen bonds (Hydrogen bond occupancy exceeding 10%).
