# Supplementary material for: Unraveling the Synergistic Inhibition of Human Maltase–Glucoamylase by Baicalein and Acarbose: Integrated Pharmacodynamics and Computational Insights
Source: Pharmaceuticals (Basel). 2026 Aug 1;19(8):1215. doi: 10.3390/ph19081215 (PMC13516409; doi:10.3390/ph19081215)
Supplement: Supplementary file 1 [file pharmaceuticals-19-01215-s001.zip › pharmaceuticals-4373756-supplementary.pdf]

Supplementary Materials

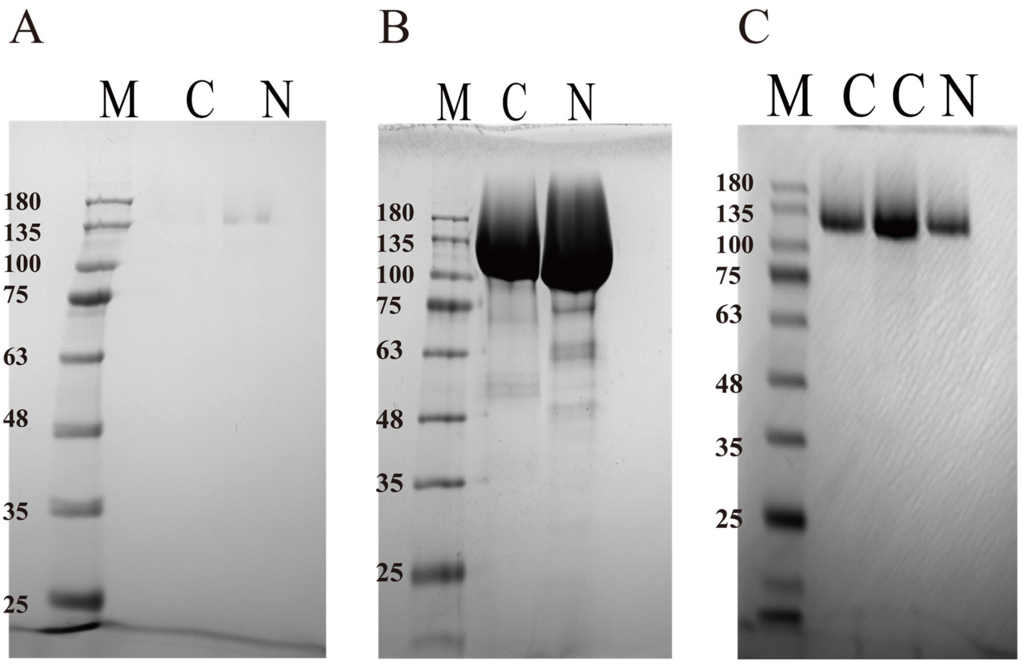

Figure S1. The SDS-PAGE analysis of MGAM-C and MGAM-N proteins. (**A**) fermentation broth; (**B**) purified by Ni Sepharose; (**C**) purified by Sephadex G-200.

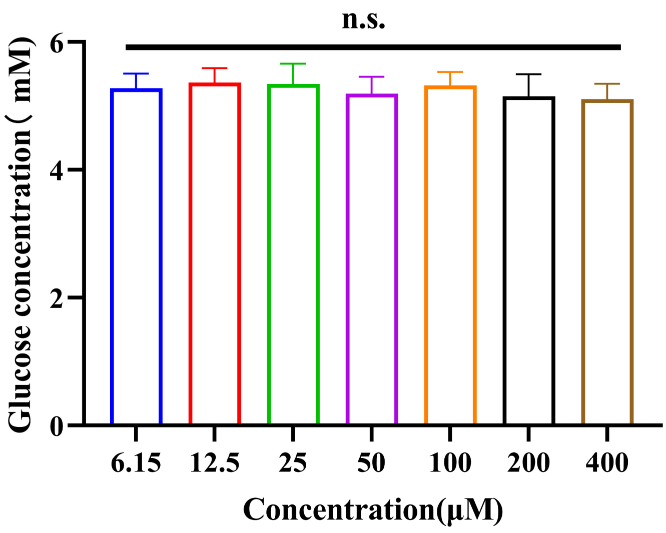

Figure S2. The impact of baicalein's antioxidant properties on the determination of glucose concentration using the glucose oxidation method.

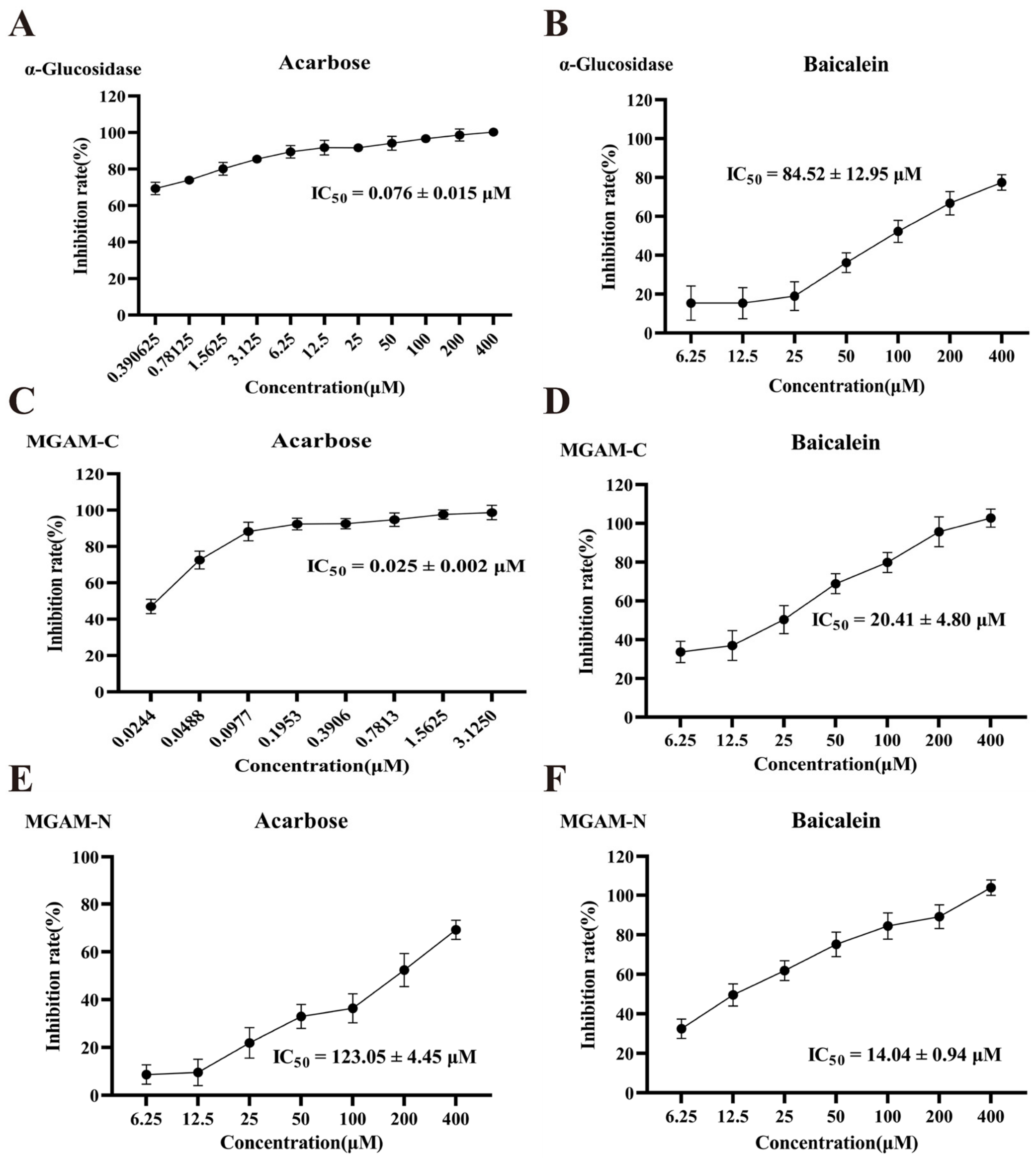

Figure S3. The inhibitory effects of acarbose and baicalein on mouse α-glucosidase (**A**,**B**), MGAM-C (**C**,**D**) and MGAM-N (**E**,**F**), respectively.

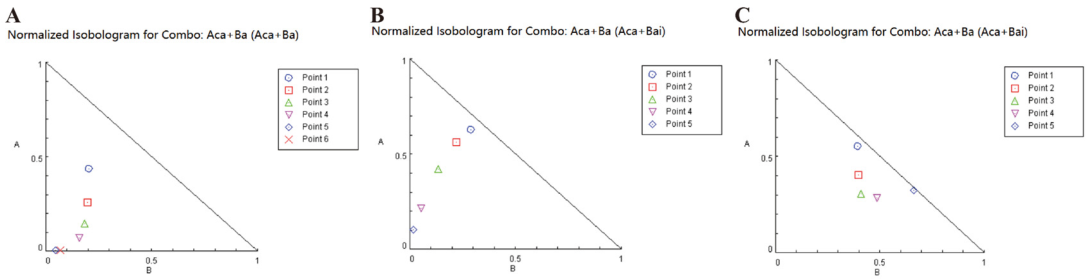

Figure S4. Normalized isobologram plots depicting the inhibitory effects of the combination of acarbose and baicalein on enzymes. (**A**) α-Glucosidase. Point 1: 0.0095 μM Acarbose + 10.5625 μM Baicalein, Point 2: 0.019 μM Acarbose + 21.125 μM Baicalein, Point 3: 0.038 μM Acarbose + 42.25 μM Baicalein, Point 4: 0.076 μM Acarbose + 84.5 μM Baicalein, Point 5: 0.152 μM Acarbose + 169 μM Baicalein, Point 6: 0.304 μM Acarbose + 338 μM Baicalein; (**B**) MGAM-C. Point 1: 0.00625 μM Acarbose + 5.1 μM Baicalein, Point 2: 0.0125 μM Acarbose + 10.2 μM Baicalein, Point 3: 0.025 μM Acarbose + 20.4 μM Baicalein, Point 4: 0.05 μM Acarbose + 40.8 μM Baicalein, Point 5: 0.1 μM Acarbose + 81.6 μM Baicalein; (**C**) MGAM-N. Point 1: 30.775 μM Acarbose + 3.5 μM Baicalein, Point 2: 61.55 μM Acarbose + 7 μM Baicalein, Point 3: 123.1 μM Acarbose + 14 μM Baicalein, Point 4: 246.2 μM Acarbose + 28 μM Baicalein, Point 5: 492.4 μM Acarbose + 56 μM Baicalein

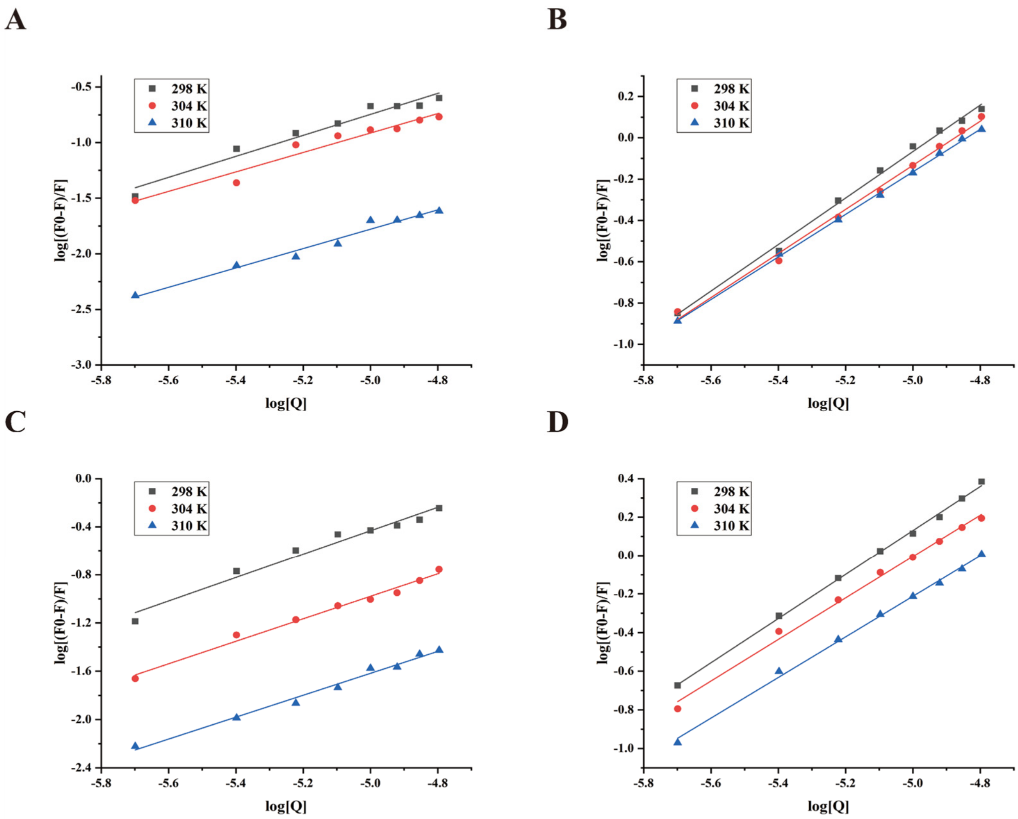

Figure S5. Double-logarithmic plot of MGAM-C. (**A**) MGAM-C_Acarbose; (**B**) MGAM-C_Baicalein; (**C**) MGAM-C_Baicalein_Acarbose; (**D**) MGAM-C_Acarbose_Baicalein.

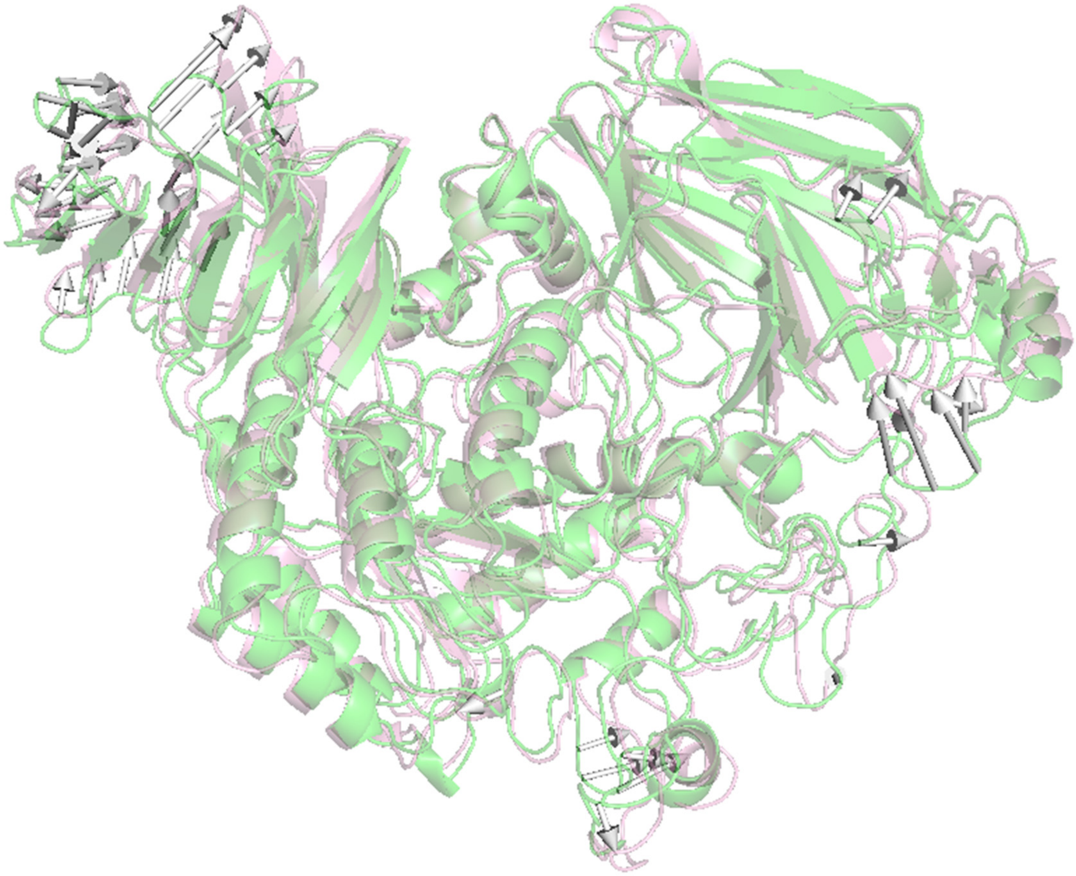

Figure S6. The direction of motion of the free 3TON main chain during molecular dynamics simulations.

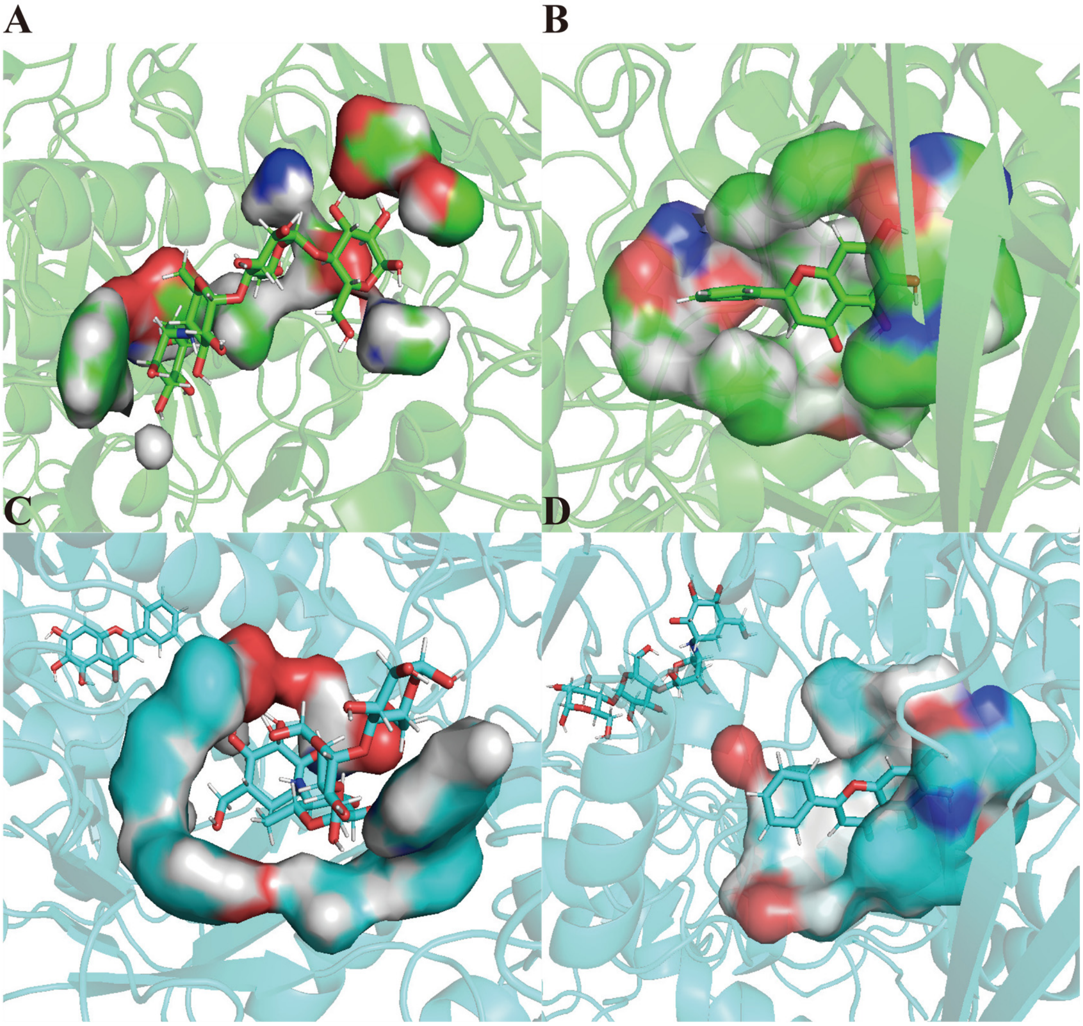

Figure S7. The binding pockets of the inhibitors on 3TON after MD (The binding pocket comprises atoms within a 4 Å radius around the molecule.). (**A**) Acarbose on 3TON_Acarbose; (**B**) Baicalein on 3TON_Baicalein; (**C**) Acarbose on 3TON_Baicalein_Acarbose complex; (**D**) Baicalein on 3TON_Acarbose_Baicalein complex.

Table S1. Individual inhibition of baicalein and acarbose against mouse α-glucosidase and recombinant MGAM.

	Inhibition rate (%) [a]	
	**Baicalein**	**Acarbose**
α-Glucosidase	77.49 ± 3.55	100.27 ± 1.64
MGAM-C	102.73 ± 4.65	98.32 ± 1.63
MGAM-N	103.84 ± 3.88	69.20 ± 4.01

[a]Inhibition rate by 400 μM inhibitors. The values shown are the averages of triplicate assays.

Table S2. Kinetic parameters for MGAM of baicalein.

	**Concentration (μM)**	V_{max} **(△OD/min)**	K_m **(mg/mL)**	K_i **(μM)**
Baicalein	0	0.0145	1.1706	14.59
	10.2	0.0080	1.2632	
	20.4	0.0061	1.2383	

Table S3. Changes of secondary structure of baicalein, acarbose and combination when acting on MGAM-C.

	α-helix (%)	β-sheet (%)	β-turn (%)	Random coil (%)
MGAM-C	9.93%	34.36%	18.27%	37.44%
Baicalein	9.96%	37.08%	16.96%	35.90%
Acarbose	10.00%	36.04%	17.13%	36.83%
Combination 1[a]	12.67%	36.53%	15.84%	34.85%
Combination 2[a]	13.15%	34.86%	16.53%	35.46%
Combination 3[a]	12.95%	34.76%	16.73%	35.56%

[a]Combination 1: Simultaneous addition of acarbose and baicalein to MGAM-C; Combination 2: Pre-incubation of MGAM-C with baicalein, followed by titration with acarbose; Combination 3: Pre-incubation of MGAM-C with acarbose, followed by titration with baicalein.

Table S4. Details of molecular docking analysis between inhibitor to 3TON.

Protein_ligand	Binding energy (kcal/mol)	Number of H-bonds	Amino acids residues	
			**Hydrogen interactions**	**Hydrophobic interactions**
3TON_Acarbose	-4.6	10	Arg1156(O16: 2.73 Å), Asp1157(O11: 3.20 Å), Gln1158(O16: 3.06 Å, O17: 3.10 Å), Lys1164(O17: 3.21 Å), Ser1452(O15: 2.95 Å), Lys1460(O7: 2.60 Å, O16: 3.19 Å), Asp1526(O13: 2.99 Å, O18: 2.84 Å)	Trp1355, Trp1369, Met1421, Phe1427, Glu1451, Phe1559, Phe1560
3TON_Baicalein	-9.3	7	Thr1621(O2: 2.71 Å, O5: 2.93 Å), Ile1716(O3: 2.69 Å, O-O4: 3.00 Å, N-O4: 3.25 Å), Trp1749(O2: 2.79 Å, O3: 3.17 Å)	Tyr1618, Leu1622, Lys1625, Glu1629, Val1631, Pro1658, Tyr1715, Pro1718, Gly1748
3TON_Baicalein_Acarbose	-4.8	10	Arg1156(O16: 2.75 Å), Asp1157(O11: 3.17 Å), Gln1158(O16: 3.05 Å, O17: 3.08 Å), Lys1164(O17: 3.06 Å), Ser1452(O15: 2.98 Å), Lys1460(O7: 2.56 Å, O16: 3.17), Asp1526(O13: 2.97, O18: 3.01 Å)	Trp1355, Trp1369, Met1421, Phe1427, Glu1451, Phe1559, Phe1560
3TON_Acarbose_Baicalein	-9.4	7	Thr1621(O2: 2.70 Å, O5: 2.90 Å), Ile1716(O3: 2.69 Å, O-O4: 3.04 Å, N-O4: 3.27 Å), Trp1749(O2: 2.81 Å, O3: 3.17 Å)	Tyr1618, Leu1622, Lys1625, Glu1629, Val1631, Pro1658, Tyr1715, Pro1718, Gly1748

Table S5. Occupancy percentages of hydrogen bonds (Hydrogen bond occupancy exceeding 10%).

	Label	Percent occupancy (%)
3TON_Acarbose	Ser1452@OG-HG...Aca@O12	37.942
	Arg1455@NH1-HH12...Aca@O9	12.041
	Arg1455@NH2-HH22...Aca@O9	13.529
	Aca@O12-H37...Gln1158@OE1	43.096
	Aca@O13-H38...Asp1526@OD1	11.695
	Aca@O14-H39...Phe1427@O	14.569
	Aca@O15-H40...Glu1451@O	30.836
	Aca@O17-H42...Asp1157@O	41.313
3TON_Baicalein_Acarbose	Asn1429@ND2-HD22....Aca@O15	10.389
	Arg1510@NH1-HH12...Aca@O10	26.756
	Arg1510@NH1-HH12...Aca@O11	14.073
	Aca@O11-H36...Asp1526@OD1	60.203
	Aca@O12-H37...Asp1370@OD1	27.351
	Aca@O12-H37...Asp1370@OD2	24.289
	Aca@O13-H38...Asp1526@OD1	62.819
	Aca@O15-H40...Asp1370@OD1	27.886
	Aca@O15-H40...Asp1370@OD2	19.898
	Aca@O16-H41...Asp1370@OD2	10.892
3TON_Baicalein	Trp1749@N-H... Bai@O3	79.722
	Bai@O3-H9...Trp1749@O	61.639
	Bai@O4-H10...Ile1716@O	86.925
3TON_Acarbose_Baicalein	1749Trp@N-H... Bai@O3	67.062
	Bai@O3-H9...1749Trp@O	46.912
	Bai@O4-H10...Ile1716@O	84.447
